# Unlocking the potential of ‘Egusi’ melon (*Colocynthis citrullus* L.) as a crop for biotechnological improvement

**DOI:** 10.3389/fpls.2025.1547157

**Published:** 2025-03-20

**Authors:** Aliya Fathima Anwar, Peter Nkachukwu Chukwurah, Erick Amombo, Salma Mouhib, Valentine Otang Ntui

**Affiliations:** ^1^ African Genome Center, University Mohammed VI Polytechnic, Ben Guerir, Morocco; ^2^ African Sustainable Agriculture Research Institute, University Mohammed VI Polytechnic, Laayounne, Morocco

**Keywords:** abiotic and biotic stresses, biodiesel, ‘Egusi’ genomics, genome editing, melon, proteomics, tissue culture, transcriptomics

## Abstract

‘Egusi’ melon (*Colocynthis citrullus* L.) plays a critical role in food security and potential biofuel production in West Africa. Its seeds are valued for both their nutritional and potential industrial applications, especially in biodiesel production. However, the crop faces significant challenges, including the impacts of climate change, water scarcity, declining arable land, and increased pressure from pests and diseases. These challenges threaten the stability of ‘Egusi’ production and may hinder its ability to meet future demand. To address these issues, there is a growing need to complement conventional breeding methods with biotechnological approaches. Molecular approaches; including genomics, transcriptomics, proteomics, and metabolomics; have been utilized for the improvement of several cucurbit species. However, information on molecular breeding of ‘Egusi’ is very limited. The current review focuses on ‘Egusi’ melon, its biology, uses, and factors affecting its improvement, and highlights critical knowledge gaps in the molecular breeding of ‘Egusi’. The review also examines the potential of omics technologies and outlines the importance of genetic transformation and genome editing methods such as CRISPR that could drive the development of more resilient and high-yielding ‘Egusi’varieties that will contribute to sustainability and profitability of ‘Egusi’ farming.

## 1 Introduction

**Figure 1:**
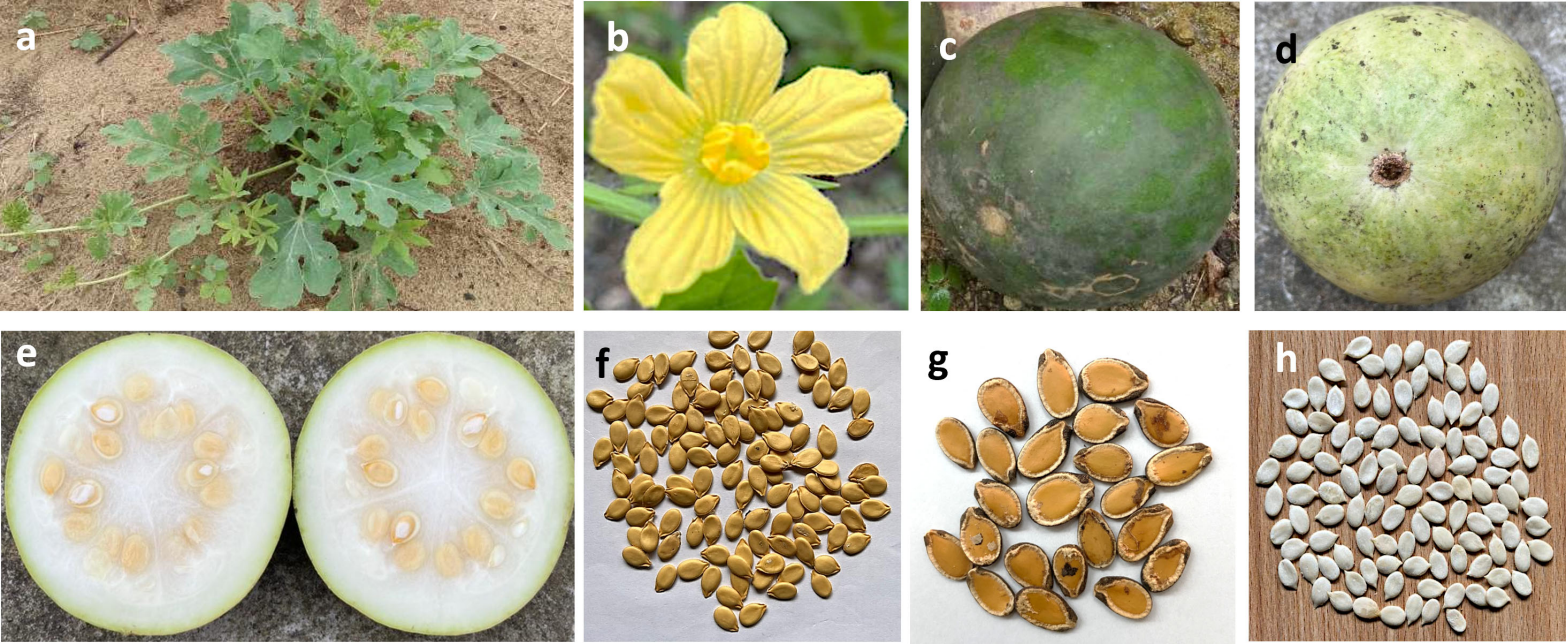
Different structures of ‘Egusi’ melon plant. **(a)** ‘Egusi’ plant in the field. **(b)** ‘Egusi’ flowers. **(c, d)** ‘Egusi’ fruits. **(e)** Fruit flesh containing ‘‘Egusi’’ seeds. **(f, g)** unshelled seeds. **(h)** shelled seeds.

‘Egusi’ (Yoruba) (also known as Agusi, Egwusi, Ohue, Ikpan, Nkon, Ikon, Agushi or Mbíka) is one of the cultivated melon species of the Cucurbitaceae (gourd) family indigenous to West Africa (Paris, 2015). It is a non-climbing creeping vine plant with deeply lobed, pinnately dissected and alternately arranged leaves (**Figure 1a**) (National Research Council, 2006). Flowers are yellow (**Figure 1b**), monoecious (male and female flowers are on the same inflorescence), and solitary, while the green spherical fruits (**Figures 1c, d**) resemble watermelon on the outside and could be easily confused as one. However, the fruit flesh (**Figure 1e**) is hard, bland, bitter, and white, and contains numerous white, flattened, narrow pulp covered with soft mucilaginous seed coats and varying in size and thickness (**Figures 1f, g**) (National Research Council, 2006). The shelled seeds are white (**Figure 1h**). ‘Egusi’ melon is categorized as an orphan/lost crop (National Research Council, 2006), as well as a neglected and underutilized species (Achigan-Dako et al., 2008), with the ability to thrive in harsh environmental conditions and with a long history of providing nutrition especially to many African communities, but without adequate research attention. It is believed to have a close relationship with the dessert watermelon (*Citrullus lanatus*), and both have been proposed to share the same genus (*Citrullus*) or have possibly been derived from same ancestral population (Guo et al., 2019). A recent investigation by Pérez-Escobar et al. (2022) into the early history of watermelon (*Citrullus lanatus*) by genome sequencing of up to 6000- year-old *Citrullus* seeds traced much of the nuclear genome of the ancient seeds to west African seed-use “Egusi”-type watermelon (*Citrullus mucosospermus*) rather than the domesticated pulp-use watermelon (*Citrullus lanatus*).

Disagreement exists in the literature on the correct botanical nomenclature of ‘Egusi’ melon possibly due to the striking phenotypic resemblance among the cucurbits. Accordingly, different botanical names exist for ‘Egusi’ melon including *Colocynthis citrullus* L. (Oji et al., 1999; Uruakpa and Aluko, 2004; Ntui and Uyoh, 2004, 2005, Ntui et al., 2009; Giwa et al., 2010; Brisibe et al., 2011; Egwim et al., 2015; Nwakaudu et al., 2017; Arthur et al., 2020), *Citrullus lanatus* (National Research Council, 2006), *Citrullus lanatus* subsp. *mucosospermus* or *Citrullus mucosospermus* (Achigan-Dako et al., 2008; Olubi et al., 2021; Pérez-Escobar et al., 2022), *Cucumeropsis mannii* (Burkill, 1985; Fokou et al., 2009; Essien et al., 2012; Opoku-Boahen et al., 2013; Azuaga et al., 2018; Okwundu et al., 2021; Nwoke et al., 2023). The National Research Council (2006), presented ‘Egusi’ melon generally as seeds of a type of watermelon - *Citrullus lanatus* which is botanically synonymous with *Colocynthis citrullus* L., but recognized however that *Cucumeropsis mannii* may be the main ‘Egusi’ consumed in West Africa. Nwoke et al. (2023) posited that the generally recognized ‘Egusi’ cultivated in Nigeria (the world’s largest melon producer and from where the local name ‘Egusi’ was derived) is the *Cucumeropsis mannii* and is commonly mistaken for *Citrullus lanatus* - a wild species of watermelon whose dehulled seeds are not white as the typical edible white-seeded ‘Egusi’ melon, and whose wet un-dehulled seeds resembles typical watermelon seeds. Taken together, both arguments seem more strongly in favor of *Cucumeropsis mannii* as the African seed-type ‘Egusi’ melon. However, in the melon genome hub (CuGenDBv2) *Citrullus mucosospermus* is presented as the botanical name of ‘Egusi’ obtained from Nigeria (Yu et al., 2023). While it might be difficult to settle the existing nomenclature argument, it is worthwhile at least to clearly define the features of ‘Egusi’ melon used in any study with clear pictures for a better appreciation of the crop under study.

**Figure 2:**
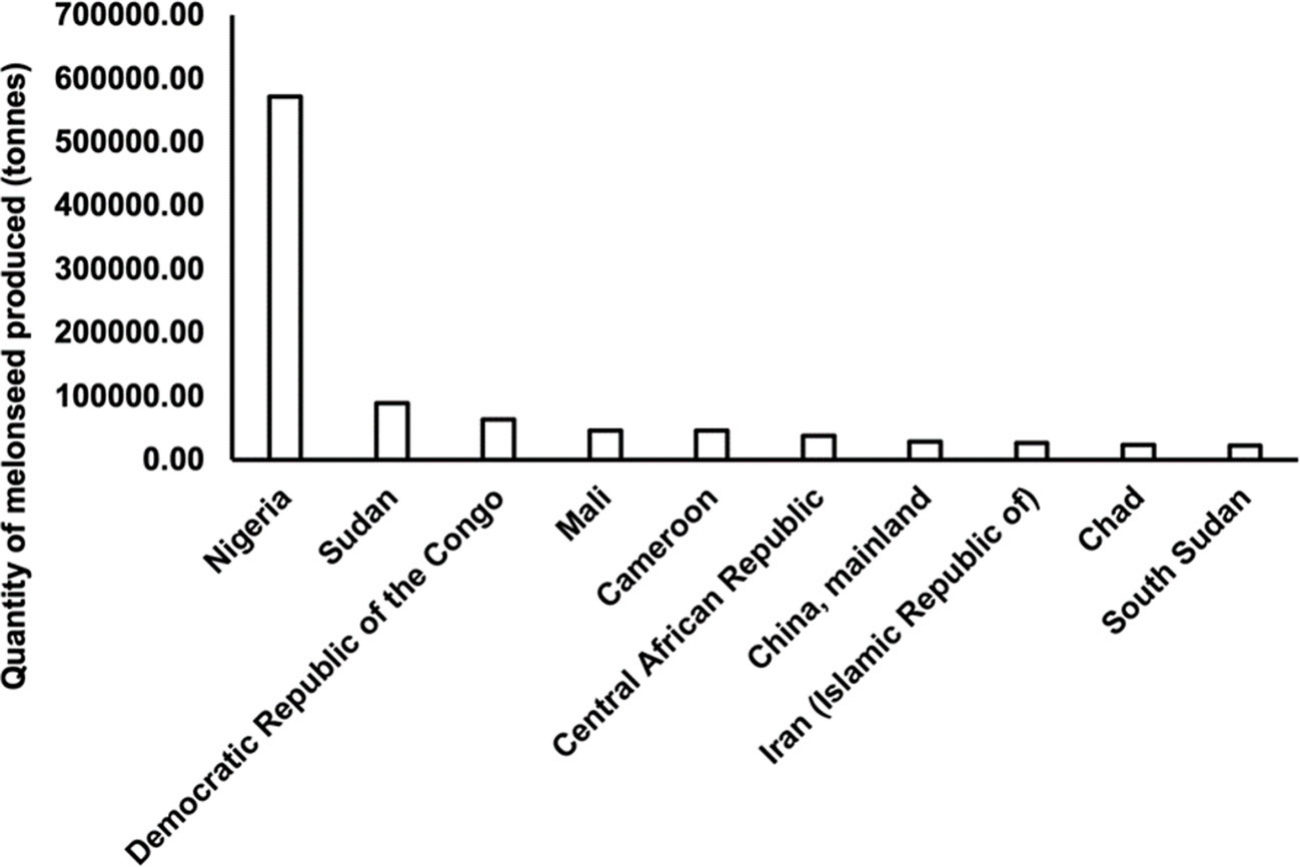
Top ten producers of melon seeds in 2022. (Source: FAOSTAT 2022, retrieved June 16, 2024).

‘Egusi’ is mostly grown in Nigeria, Burkina Faso, Togo, Ghana, Côte d’Ivoire, Benin, Mali, and Cameroon (National Research Council, 2006). Accurate data on the production of ‘Egusi’ is lacking. However, FAO statistics show that global melon seed cultivation, which includes all cucurbit seeds, increased from slightly under 500,000 hectares in 1961 to over 1.8 million hectares in 2022, and production from slightly less than 300,000 tons in 1961 to over 900,000 tons in 2022 (FAO, 2022). Nigeria ranks as the highest melon seed producer with an estimated 5.7 x 10^5^ tons (**Figure 2**), a greater percentage being ‘Egusi’ seeds.

In this review, we focus on the uses and challenges of ‘Egusi’ production in West Africa. We summarized the biotechnology tools applied for the improvement of ‘Egusi’. Then, we discussed the potential applications of advanced techniques such omics as well as genome editing in ‘Egusi’ breeding. Advances made in CRISPR/Cas9 genome editing, regulatory and ethical aspects associated with “Egusi” genome editing were also considered. We conclude that omics, genetic transformation and genome editing offer a more sustainable approach for the improvement of the crop.

## 2 Economic importance of ‘Egusi’ melon (including potential for biodiesel production)

‘Egusi’ melon is primarily produced for its high protein and oil-containing seeds, used as food in many West African communities. Proximate analysis of full-fat and defatted ‘Egusi’ seed flour by Nwoke et al. (2023) revealed fat content (48.34 ~ 49.83 and 0.23 ~ 0.24 g/100g, respectively), protein (35.46 ~ 37.45 and 66.44 ~ 69.04 g/100g, respectively), and carbohydrate content (2.63 ~ 3.43 and 12.36 ~ 16.26 g/100g, respectively). As food, the raw dehulled seeds are usually processed in various forms including drying, roasting, toasting, and fermentation before use as snacks, in the preparation of soups, animal feed, etc. (**Table 1**). Young leaves and seed husks have less application compared to the seeds but have been reported to have some value as food and industrial feedstock respectively (**Table 1**). However, aside from its use as food, the second most important potential application of ‘Egusi’ seeds is arguably the use of the seed oil as feedstock for renewable energy (biodiesel) production. Analysis of ‘Egusi’ seed oil showed 53.5% lipids (Okwundu et al., 2021), while fatty acid composition of the seed oil showed high amounts of linoleic and oleic acids (Akubor and Ogbadu, 2003; Olubi et al., 2019). Interestingly, defatted ‘Egusi’ seed flour has been shown to contain even more protein and carbohydrate than full-fat flour (Nwoke et al., 2023), thus extraction of its seed oil for use in biodiesel production may not significantly reduce the full potential of the residual cake as food. Alternatively, to reduce pressure on the use of edible ‘Egusi’ seed oil as sole feedstock, it can be mixed with other non-edible oils to obtain hybrid oil feedstocks – a practice that is currently emphasized for biodiesel synthesis given the advantages of cost reduction and improved fuel quality (Brahma et al., 2022).

**Table 1:** Uses of ‘Egusi’ Melon.

Plant part	Processing method	Use	References
**Seed**	Dried & ground into flour (non-defatted)	Soup condiment & thickener	Giwa et al., 2010; Egwim et al., 2015; Arthur et al., 2020
	Dry roasted	Eaten as snack	National Research Council, 2006
	Dry roasted & processed into paste	As spread on bread; food additive	National Research Council, 2006
	Toasted, dehulled & water blended	Base for beverage drink	Akubor and Obasi, 2019
	Fermented	Local spice (“ogiri-egusi”), flavor condiment	Uzogara et al., 1990
	Fermented & cooked	As sole protein in poultry feed	Oloyede et al., 2004
	Defatted seed flour (residual cake)	Meat substitute; ready-to-eat instant soup	Schippers, 2000; Olubi et al., 2021
	Extracted seed oil	Potential food protein supplement/fortifier	Okwundu et al., 2021
		Local snack	Ilodibia et al., 2014
		Animal (cattle) feed	Ilodibia et al., 2014
		Cooking & salad oil	National Research Council, 2006
		Heavy metal removal agent	Okwundu et al., 2021
		Lowers total cholesterol levels in rats	Oladapo et al., 2017
		Pesticide against post-harvest weevils	Nzelu and Okonkwo, 2016
		Biodiesel feedstock	Giwa et al., 2010; Bello and Makanju, 2011; Muhammad et al., 2013; Opoku-Boahen et al., 2013; Hairuddin et al., 2019; Okwundu et al., 2021
**Leaves**	Cooked	Eaten as potherb	National Research Council, 2006
**Seed husk (shell)**	Rinsed & dried	Biodiesel feedstock	Longjan and Dehouche, 2018
	Pulverized	Pyrolysis feedstock	Nyakuma, 2017
	Burning & milling to ash	Material (alloy) reinforcement	Suleiman et al., 2018
	Washed, dried & milled	Isolation of nanocrystalline cellulose	Ogundare et al., 2021

Transesterification is currently the most used method for producing eco-friendly biodiesel from vegetable oils. It involves the reaction of the vegetable oil (triglycerides) with low molecular weight primary alcohols (mostly methanol) (Kouzu and Hidaka, 2012), in the presence of a catalyst (heterogenous, homogenous, or enzyme) (Brahma et al., 2022), to produce biodiesels (called fatty acid alkyl esters), and glycerol obtained as by-product. Of the catalysis systems used in transesterification, natural heterogenous catalysts are considered more environmentally friendly with additional advantages of being easily separated from the final product and possibly re-used (Gaide et al., 2021). Transesterification of ‘Egusi’ seed oil has been achieved using both homogenous (Giwa et al., 2010; Opoku-Boahen et al., 2013) and heterogenous (Okwundu et al., 2021) catalysts with biodiesel yield ranging from 82~86%. A comparison of biodiesel yield from ‘Egusi’ oil transesterification with some other edible and non-edible vegetable oils using natural heterogenous catalysts is presented in **Table 2**. Although biodiesel yield from ‘Egusi’ seed oil falls slightly lower than that of known oil crops like *Jatropha*, it is worth noting that research on optimizing biodiesel production in the orphan crop ‘Egusi’ has not been carried out as much as has been done on *Jatropha*. It is thus feasible that higher biodiesel yields from ‘Egusi’ seed oil could be achieved with increased research attention on optimization of transesterification and feedstock parameters. Molecular breeding for production of high-performance ‘Egusi’ crops by traditional genetic engineering and/or genome editing are viable pathways for the optimization of biodiesel production from ‘Egusi’ melon as ‘Egusi’ feedstock biomass. Such breeding efforts, among other targets, could be focused on producing feedstock with higher seed oil content through strategies such as metabolic engineering, increasing oil accumulation in non-seed vegetative tissues such as leaves, and improving overall resistance of the crop to abiotic and biotic stresses (Wan et al., 2017).

**Table 2:** Comparison of “Egusi” seed oil with oil from other seed crops for biodiesel production using natural heterogeneous catalyst systems.

Seed oil	Catalyst used	Transesterification parameters for optimal biodiesel yield			Biodiesel yield (%)	Reference
		Alcohol: oil molar ratio	Optimal temp. (°C)	Optimal time (min)		
‘Egusi’ melon (C*olocynthis citrullus*)	decalcified shell of desert snail	MeOH: oil (9:1)	60	180	86.00	Okwundu et al., 2021
Jatropha (*Jatropha curcas*)	nano-CaO from *P*. *erosa* seashells	MeOH: oil (5.15:1)		133.1	98.54	Reddy et al., 2016
Soybean (*Glycine max*)	nano-CaO from chicken eggshell	MeOH: oil (6:1)	65	180	85.83	Santos et al., 2019
Rapeseed (*Brassica napus*)	dolomite	MeOH: oil (11.94:1)	64	300	98.05	Gaide et al., 2021
Castor plant (*Ricinus communis*)	calcined bovine bones	MeOH: oil (6:1)	60	60	95.12	Owolabi et al., 2020
Date seed (*Phoenix dactylifera*)	waste camel bones	EtOH: oil (7:1)	75	180	89.00	Alsaiari et al., 2023
Palm oil (*Elaeis guineensis*)	calcined mussel shell	MeOH: oil (9:1)	65	240	97.23	Buasri et al., 2013
Sunflower (*Helianthus annuus*)	clinoptilolite (a natural zeolite)	MeOH: oil (7:1)	65	180	97.00	Tahvildari et al., 2014
Cotton seed (*Gossypium hirsutum*)	CaO derived from eggshell	MeOH: oil (9:1)	60	180	98.03	da Silva Castro et al., 2019
Canola (*Brassica napus*)	CaO derived from eggshell	MeOH: oil (9:1)	50	180	97.60	Khabiti et al., 2021
Neem (*Azadirachta indica*)	waste cow bone	MeOH: oil (9:1)	59	240	87.04	Oke et al., 2021
Coconut (C*ocos nucifera*)	calcined scallop shell waste	MeOH: oil (12:1)	60	120	91.70	Hidayat et al., 2022
Camelina (*Camelina sativa*)	waste eggshells, lobster shells	MeOH: oil (12:1)	65	180	97.20, 90.00	Hangun-Balkir, 2016
Flaxseed (*Linum usitatissimum*)	calcined *Musa acuminata* peels	MeOH: oil (11:1)	65	51.42	96.50	Etim et al., 2022

## 3 Challenges in ‘Egusi’ melon production

### 3.1 Biotic stresses

‘Egusi’ is highly susceptible to a variety of pathogens that can severely affect its growth and productivity. Despite its significance, there is a paucity of information and limited updates regarding critical diseases impacting ‘Egusi’ melon, particularly concerning the distribution and status of blight. This knowledge gap poses challenges for effective disease management strategies. In this section, we review the existing literature on the biotic stresses affecting ‘Egusi’ melon and their causal agents. Furthermore, we evaluate the current management solutions that have been employed.

One of the most prevalent diseases is mosaic virus, particularly caused by the Papaya ringspot virus. Infected plants exhibit characteristic symptoms such as chlorotic mottling, leaf deformation, stunted vegetative growth, and malformed fruits. The virus is predominantly vectored by aphids and other sap-sucking insects and can also be seed-borne (Naeimifar et al., 2014). Integrated pest management strategies, including aphid control and the use of certified virus-free seeds, are being applied to mitigating its transmission.

Another common disease affecting ‘Egusi’ melon is powdery mildew, which is primarily caused by the fungal species *Erysiphe cichoracearum* and *Podosphaera xanthii*. The disease is typified by a superficial white mycelial growth on the aerial parts of the plant, leading to chlorosis and necrosis of leaves, which, in turn, limits the plant’s photosynthetic capacity. Severe infections result in significant yield losses due to reduced vegetative vigor (Lebeda et al., 2024). Fungicide application and adequate plant spacing to improve air circulation are commonly used management practices. Anthracnose, caused by *Colletotrichum lagenarium*, is another significant fungal disease in ‘Egusi’ melon cultivation. It is characterized by necrotic, water-soaked lesions on foliage, stems, and fruits, which eventually develop into sunken, dark necrotic areas. This pathogen thrives under high humidity conditions and can lead to extensive fruit rotting and crop loss. Current management strategies include the use of resistant cultivars, application of fungicides, and crop rotation to reduce pathogen persistence in the field (Patel et al., 2023). Downy mildew, caused by the oomycete *Pseudoperonospora cubensis*, poses a serious threat to ‘Egusi’ melon. It manifests as yellow, angular chlorotic lesions on the adaxial leaf surfaces, with corresponding greyish sporulation on the abaxial side. If unchecked, downy mildew can cause premature defoliation, severely compromising photosynthesis, and overall plant vigor, resulting in yield decline (Call et al., 2011).

A particularly destructive disease is Fusarium wilt, caused by the soil-borne fungus *Fusarium oxysporum f.* sp. *niveum*. This pathogen colonizes the vascular tissues of the plant, inducing systemic wilting, chlorosis of the lower leaves, and eventual plant death (Pal et al., 2023). The pathogen’s ability to survive in the soil for extended periods complicates control efforts. Disease management relies on the use of resistant varieties and the implementation of crop rotation with non-host species to limit soil pathogen load (Ponsankar et al., 2023). Root rot, primarily caused by soil-borne pathogens such as *Pythium* and *Rhizoctonia* spp., is another significant constraint in ‘Egusi’ melon production. Infected plants exhibit root necrosis, which impairs nutrient and water uptake, leading to stunted growth, foliar chlorosis, wilting, and plant death in severe cases (Pane et al., 2020).

**Figure 3:**
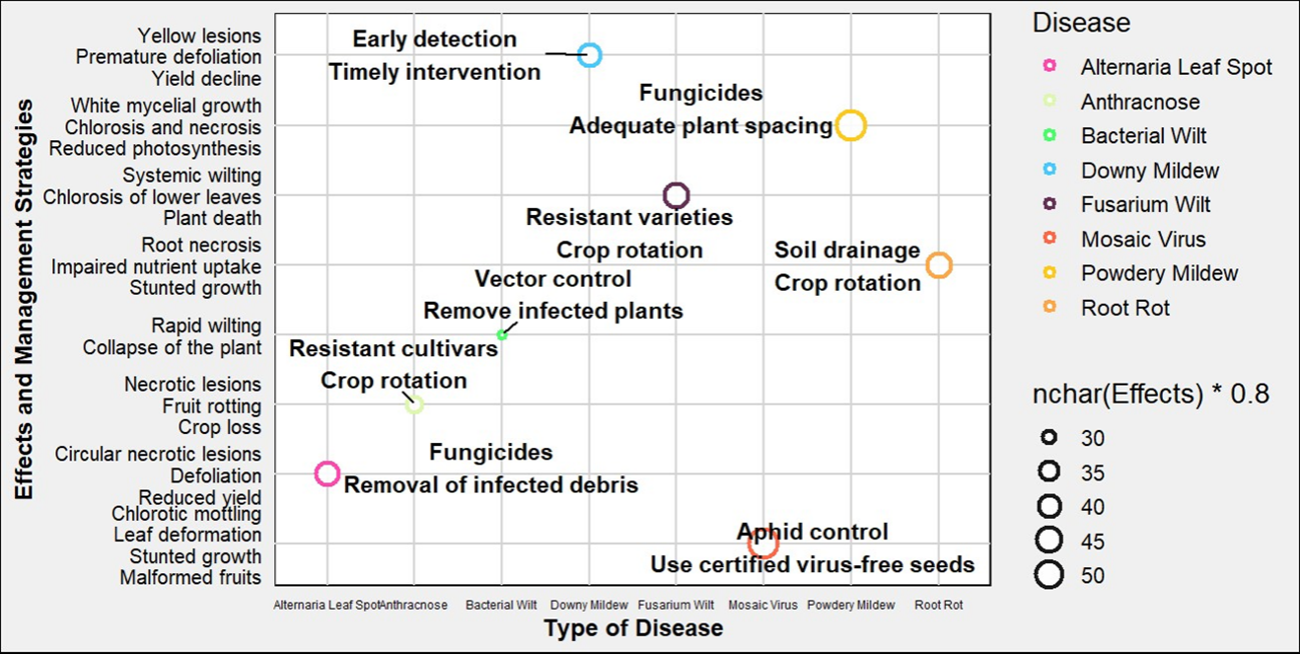
Schematic representation of various plant diseases in ‘Egusi’melon, their target pathogens, and symptoms. Each point represents a disease, colored according to its associated pathogen. Management strategies are annotated beside each point.

Alternaria leaf spot, caused by *Alternaria cucumerina*, is also a notable foliar disease in ‘Egusi’ melon. It presents as small, circular, necrotic lesions with concentric ring patterns on the leaves, which can coalesce, leading to extensive leaf necrosis and defoliation. This disease significantly impairs the plant’s photosynthetic ability, leading to reduced yield (Ntui et al., 2010b). The use of fungicides and removal of infected plant debris can help mitigate disease spread. Lastly, bacterial wilt, caused by *Erwinia tracheiphila*, is a vascular disease transmitted by cucumber beetles. The pathogen causes rapid wilting and collapse of the plant due to blockage of water-conducting tissues (Al-Shuaibi et al., 2023). Effective management involves vector control and the removal of infected plants to limit disease dissemination (**Figure 3**).

Many of these approaches, however, are not sustainable in the long term, often due to environmental consequences or the emergence of resistant pathogen strains. For instance, while management of these diseases through pesticides has been effective, there have been the risk of crop resistance. For example, resistance breakdown in cucurbit crops and the emergence of fungicide-resistant strains have been reported calling for more sustainable measures (Blum et al., 2011; Wallace et al., 2017). This highlights the urgent need for innovative disease management strategies. We propose the exploration of more precise methodologies, such as genome editing, by targeting host-pathogen interaction to enhance disease resistance in ‘Egusi’ melon.

### 3.2 Abiotic stresses

Drought, salt, and heat stresses typical of African climates are among the most formidable constraints impeding ‘Egusi’ melon production. While ‘Egusi’ melon flourishes in mesophilic conditions, excessive thermal exposure can induce stress, particularly during the critical reproductive phase of pollination. The delicate thermal balance is paramount; while elevated temperatures promote vegetative growth, prolonged exposure to high thermal regimes can precipitate reduced fruit set and diminished fruit quality. Conversely, lower ambient temperatures can delay germination and truncate the growing season, thereby limiting the crop’s productive potential. Although the species is adapted to perform optimally well under semi-arid climatic conditions, it exhibits heightened sensitivity to hydric stress, particularly during pivotal phenological stages such as flowering and fruiting (Abushamleh et al., 2022). In addition, salinity similarly presents a significant agronomic challenge. In regions characterized by suboptimal irrigation practices, excessive salt accumulation in the edaphic environment can inhibit seed germination and root ontogeny. Saline conditions disrupted ‘Egusi’ melons capacity for nutrient uptake, manifesting as chlorotic foliage and overall physiological decline (Abushamleh et al., 2022).

**Figure 4:**
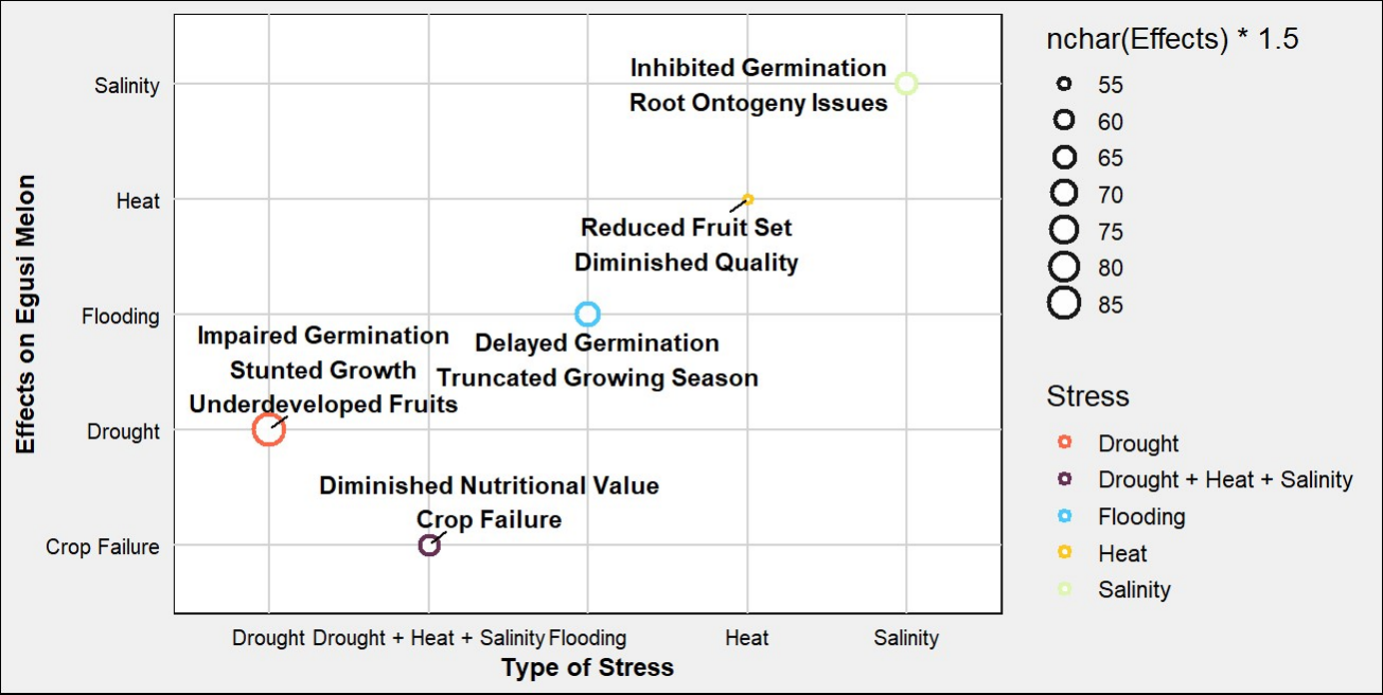
Schematic representation illustrates the effects of various environmental stresses on ‘Egusi’ melon. Each point represents an abiotic stress, colored according to its associated effects.

The starting point for addressing abiotic stress tolerance is by looking at the biochemical pathways during response. ‘Egusi’ melon has been observed to modulate photosynthetic and biochemical pathways to enhance stress resilience and sustain growth under sub-optimal thermal conditions (Elnaggar et al., 2024). Under water stress, ‘Egusi’ melon plants exhibited impaired germination, stunted vegetative growth, and the production of underdeveloped fruits. The ramifications of water stress extend beyond quantitative yield losses; the quality of the seeds is compromised, resulting in diminished nutritional value, and reduced economic viability for producers (Amali et al., 2013). **Figure 4** highlights the effects of drought, heat, salinity, and flooding impact on the growth and development of the crop. Drought conditions lead to impaired germination, stunted growth, and the production of underdeveloped fruits. In contrast, exposure to excessive heat results in reduced fruit set and diminished quality. Salinity affects the plant by inhibiting germination and causing issues with root development, while flooding can delay germination and truncate the growing season. Ultimately, the cumulative impact of these stresses can lead to crop failure, reflecting the combined detrimental effects of drought, heat, and salinity. This not only compromises the nutritional value of the fruit but also threatens the viability of the crop, posing significant challenges for producers. The interplay between abiotic stresses and pest or disease susceptibility adds another layer of complexity. Stressed plants are often more attractive to pests, which can exploit their weakened state. Drought-stressed ‘Egusi’ melons, for instance, may attract aphids, while fungal infection can take hold more easily in plants already battling nutrient deficiencies (Rahman et al., 2024). To safeguard ‘Egusi’ melon production against these abiotic stresses, several strategies can be employed. Molecular breeding programs aimed at developing stress-tolerant varieties hold significant promise. This requires more studies on the biochemical pathways involved in tolerance mechanisms. For instance, it has been reported that ‘Egusi’ melon exhibits adaptive responses to stress through the accumulation of citrulline and the transcriptional upregulation of genes implicated in drought tolerance such as APX and Type-2 metallothionein, as indicators of salinity tolerance (Song Q. et al., 2020). Emerging evidence indicates that transcription factors regulating the expression of these functional genes play a critical role in orchestrating drought and salinity stress responses and adaptations (Malambane et al., 2023) and may serve as targets for improvement.

## 4 Omics strategies for enhancing ‘Egusi’ production

The integration of omics strategies can provide a comprehensive understanding of genomic regions that encode economically important traits and key components of plant stress mechanisms, biological pathways and regulatory gene networks (Yang et al., 2021). The advent of high throughput genotyping techniques such as QTL (Quantitative Trait Loci), marker-assisted selection, whole genome sequencing, RNA-sequencing serve as an avenue for understanding the molecular mechanisms, enabling genetic manipulation to enhance breeding, resistance and overall, crop improvement (Xu et al., 2017; Kushanov et al., 2021; Roy et al., 2024). ‘Egusi’ melon has been focused for seed consumption due to its nutritive properties and high seed oil (Enujiugha et al., 2023). Nevertheless, the plant is highly susceptible to abiotic and biotic stresses (Ogunsola et al., 2020). Several genotypes of ‘Egusi’ melon have been reported to be disease resistant and studies have been conducted to improve fruit quality and yield through hybridization (Ntui and Uyoh, 2004, 2005; Ogbu et al., 2016; Orji et al., 2016). Therefore, ‘Egusi’ melons can be potential crops for breeding for yield and stress adaptation. Currently, the understanding of ‘Egusi’ crop genetic diversity may be indispensable for plant breeding and developing of improved cultivars. In this section, we summarize the application of various omics approaches including genomics, transcriptomics and proteomics as prospective strategies for enhancing melon productivity (**Table 3**).

**Table 3:** Studies on various omics approaches used in improvement of melon varieties.

Melon Variety	Approach	Outcome	Reference
Kuizilikjiz (*C. melo* L. var. *inodorus*)	Telomere to telomere Denovo genome sequence and assembly for Meta-QTL Analysis	Identification of Meta-QTL for carotenoid accumulation, flesh firmness	Wei et al., 2023
*Cucumis melo*	RNA-Seq based QTL and expression QTL analysis	QTL mapped for fruit quality- aroma, flesh color	Galpaz et al., 2018
*Cucumis melo* Parent USDA-846-1 × Top Mark Piel de Sapo × PI124112	Genotyping with SNP markers	Linkage mapping and QTL for fruit shape	Paris et al., 2008; Díaz et al., 2014
*Cucumis melo L.*	Sequenom MassARRAY	Identification of genetic variations in fruit ripening and sugar accumulation	Leida et al., 2015
*Cucumis melo* var. *acidulus*	Combined transcriptomics and metabolomics	Understanding sugar and organic acid metabolism to determine fruit quality	Cheng et al., 2022
*Cucumis melo* var. Charmono	Whole Genome Sequencing, integration of RNA-seq, Hi-C, and histone and DNA methylation marks	Chromosome level genome assembly Annotation of transposable elements, chromatin architecture,Epigenomic analysis	Pichot et al., 2022
*Cucumis melo* and 6 wild species *C. zeyheri*, *C. prophetarum*, *C. anguria*, *C. dipsaceus*, *C. myriocarpus*, *and* *C. ficifolius*.	Whole Genome Sequencing	SNP analysis, Structural variants identified	Demirci et al., 2021
*Cucumis melo subsp. melo, PIT92*	Chloroplast and Mitochondrial Genome sequencing	High proportion of nuclear DNA in revealed in organellar DNA	Rodríguez-Moreno et al., 2011
*Cucumis melo L.* var. *PI442177 (K10-1)* *Huangdanzi (K10-9)*	RNA Sequencing and Metabolomics	Transcriptomics and metabolite analysis during the response to Downy mildew (DM) disease	Ling et al., 2023
*C. melo var ameri*, *cv Flavor No. 4*	RNA Sequencing and Metabolomics	Transcriptomics of fruit development Differentially expressed genes involved in fruit ripening and development	Zhang et al., 2016
*Cucumis melo* cv. *Piel de Sapo*	Small RNA sequencing	Identification of miRNA involved in response to stress stimuli	Sanz-Carbonell et al., 2020
*Cucumis melo* spp. *melo ‘Piel de sapo cv piñonet’* *C. melo* spp. *agrestis ‘Pat 81*	Microarray analysis	Root transcriptomics in response to soil borne pathogen infection	Roig et al., 2012

### 4.1 Genomics strategies for identification of melon traits

Analysis of the relationship between phenotype and corresponding genotype using QTLs has been promising in identifying agronomic traits for improving crop yield and quality (Dogan et al., 2023). A series of studies have identified a range of QTLs in melons, with a focus on fruit traits with fruit ripening being one of the challenges associated with melon. Ripening of fruit is an important phenomenon as it promotes flavor, texture, aroma and color (Seymour and Granell, 2014). In watermelon fruit (*Citrullus lanatus * L.), a relative of ‘Egusi’ melon, the ripening behavior of the fruit shows climacteric and non-climacteric genotypes which can affect it s shelf-life (Flores et al., 2002; Beaulieu, 2005). The study conducted by Fan et al. (2000) identified the QTLs for solids concentration, rind hardness, and fruit weight. Three QTLs contributing to fruit ripening identified in climacteric and non-climacteric genotypes were found to be epistatically interacting affecting ethylene production and aroma (Santo Domingo et al., 2022). The QTLs identified from introgression lines of two European varieties climateric *cantalupensis* ‘Védrantais and non-climacteric *inodorus* ‘Piel de Sapo altered ripening, morphology and sugar content of the melon fruit (Pereira et al., 2021). Whole genome sequencing in melons has been a key tool in the development of genetic markers for breeding and the identification of genome-wide single nucleotide polymorphisms (SNPs) between different varieties (Sim et al., 2018). Constructing high density genetic maps using whole genome sequencing has been effective in identifying QTLs (Wang et al., 2012; Gardner et al., 2014; Liu et al., 2016). Recently, researchers have identified QTLs for fruit firmness in melons based on novel whole-genome single nucleotide polymorphism-based cleaved amplified polymorphic sequence (SNP-CAPS) markers (Zhang, 2020). The use of novel SNPs-based CAPS markers has further enhanced the accuracy of QTL mapping, with the potential to guide genetic engineering and molecular breeding in melons (Amanullah et al., 2018; Zhao et al., 2023). The availability of complete genomes from different melon accessions has provided a valuable reference for meta-QTL analysis (Wei et al., 2023). The published QTLs can be integrated onto these complete genomes thereby improving accuracy in identification of candidate genes, further enhancing our understanding of the genetic basis of these traits. The use of advanced genomic technology including short and long read sequencing for *de novo* assembly to create a pan-genome of parental combinations demonstrated to be a potential tool for in depth assessment of the complex horticulturally important traits (Oren et al., 2022). Therefore, overcoming the reliability of single reference for QTL analysis can be avoided in understanding the genetic basis of various traits (Tettelin and Medini, 2020). In ‘Egusi’ melons, the QTL associated with unique ‘Egusi’ seed coat trait locus (eg) and seed oil percentage have been mapped, which has been further refined to the locus which is a 398.25 Kb long region on chromosome 6, containing 30 candidate genes using SNPs identified from QTL-seq (Prothro et al., 2012; Paudel et al., 2019). Current research on identifying agronomic traits in ‘Egusi’ melon is limited. Therefore, extending findings from other melon varieties could offer valuable insights into the genetic mechanisms underlying melon traits and potentially be applied to genomics-assisted crop breeding, aimed at improving ‘Egusi’ melon productivity.

### 4.2 Transcriptomics and proteomics approaches to develop resilient ‘Egusi’ melon

Climate change is posing a threat that can result in substantial loss of crop yield or produce low quality fruit. However, these environmental changes have led to evolution and adaptation of stress tolerant plants (Lal et al., 2023). Plant response to abiotic and biotic stresses at the molecular level involves expression of transcription factors and non-coding RNAs that regulate downstream genes in developing resistance to these changes (Baillo et al., 2019). RNA sequencing has been extensively employed to characterize genes related to disease resistance in plants by providing precise data on transcript levels and their respective isoforms. Powdery mildew disease in melon is primarily caused by fungal pathogens identified as *Podosphaera xanthii, Sphaerotheca fuliginea, Sphaerotheca fusca* causing damage to melon leaves significantly reducing melon productivity (Takikawa et al., 2015). A comparative transcriptome study showed differentially expressed genes involved in powdery mildew disease resistance in melon (Zhao et al., 2022). The genes upregulated during mildew powdery caused by *P. xanthii* were among those involved in photosynthesis in correlation with observing chlorophyll fluorescence in the infected leaves (Polonio et al., 2019). This is important because chloroplasts play a crucial role in generating free radicals and producing plant defense molecules (Kretschmer et al., 2019). Further, microarray analysis of gene expression profiles can reveal extensive transcriptomic data restructured for better understanding of genes involved in disease resistance (Gonzalez-Ibeas et al., 2012). Abiotic stress caused in the presence of low-light has shown to affect fruit development and nutritional composition of watermelon (Gao et al., 2023). Salt stress has shown to alter chlorophyll fluorescence and amino acid metabolism (Song Q. et al., 2020). Transcriptomic analysis showed a plethora of genes involved in stress response that are differentially expressed including members of the transcription factors bHLH, ERF, NAC and WRKY (Li et al., 2020).

Transcriptomic approach has been used in the characterization of developing melon fruits from climacteric and non-climateric genotypes. As fruit ripening imparts sugar accumulation and therefore palatable taste, sucrose content can be measured as the marker for distinguishing ripening patterns. This correlated with the gene expression profiles in mature melon fruit with upregulation of invertase resulting in loss of sucrose in climateric genotypes and higher expression of invertase inhibitors with much stable sucrose content in non-climateric genotypes (Saladié et al., 2015). The distinct gene expression profiles for carotenoid, sugar and ethylene biosynthesis suggest different transcriptional regulation influencing fruit ripening. Additionally, a combined metabolite-gene analysis utilizing transcriptomics and metabolomics approaches can provide valuable information for genetic manipulation to improve taste and melon fruit quality (Shao et al., 2024). Investigating transcriptome gene expression patterns in non-fruit tissues of the plant additionally helps in understanding the co-expressed genes that also play a role in the fruit ripening mechanisms (Yano et al., 2018). Moreover, understanding the gene networks involved in flowering patterns can provide insights on seed formation and fruit development (Wang Z. et al., 2023).

Understanding the mechanisms that regulate plant adaptation to abiotic and biotic stresses at all levels is essential for the selection of cultivars for optimal breeding or genetic manipulation for trait improvement (Salekdeh and Komatsu, 2007; Kosová et al., 2014). The onset of advanced high throughput proteomics technologies has established a platform for analyzing quantitative protein profiles, protein-protein interactions and signaling pathways, protein localization at the subcellular level and post translational modifications (Ford et al., 2011; Uday et al., 2024). Proteome analysis is crucial because gene expression at the transcript level often does not correlate with protein profiles, primarily due to changes in post-translational modifications (Belouah et al., 2019). Series of studies have been conducted in melon cultivars that integrate proteome profile with the transcriptomic data providing deeper understanding on the complexity of molecular pathways involved in melon fruit development and ripening (Yu et al., 2022) and response to viral infection (Li et al., 2020). Proteomic analysis has provided a great advantage in understanding the stress response mechanisms in plants (Mustafa and Komatsu, 2021). Quantitative proteome profiles under water deficit conditions have shed light on drought related marker genes that cause drought tolerance in wild watermelon (Yoshimura et al., 2008; Akashi et al., 2011). The proteome and metabolome profiles during cold stress conditions have been studied in cold tolerant and sensitive cultivars of cantaloupe melon (Song W. et al., 2020). Glutathione metabolism has been found to play a significant role in detoxification reactions in response to osmotic stress caused by cold stress and promote tolerance (Song et al., 2021). Recently, grafting in melons has been shown to enhance stress tolerance, and the molecular mechanisms have been effectively studied through proteome and transcriptome analysis (Shi et al., 2019; Wang Y. et al., 2023). A number of studies have been conducted to investigate phloem proteins that are upregulated during viral infections (Gómez et al., 2005; Malter and Wolf, 2011; Serra-Soriano et al., 2015). These studies are valuable for understanding viral pathogenesis, plant-virus interactions and exploitation of these viruses can be useful in manipulating the viral genomes for virus-based vector applications in ‘Egusi’ melon.

## 5 Future prospect of using genetic modification and genome editing for ‘Egusi’ improvement

There has been some progress in the improvement of ‘Egusi’ through conventional breeding. Nevertheless, poor genetic variety and plant biology-related issues have prevented conventional breeding from being extensively utilized. For improvement of ‘Egusi’ conventional breeding needs to be complemented with genetic modification and genome editing techniques. These techniques enable the transfer of beneficial traits between different ‘Egusi’ species or within the same species, or by manipulating exogenous and endogenous genes, circumventing natural breeding limitations, thereby making them suitable for ‘Egusi’ improvement. Moreover, genes and molecules associated with defense signaling can easily be manipulated to provide resistance to pests and diseases.

### 5.1 Genetic transformation approaches for improvement of ‘Egusi’

Plant regeneration system is one of the three fundamental resources for molecular breeding and it is a prerequisite for genetic transformation of any plant species. The lack of efficient regeneration system causes limitations on the use of gene transfer technologies. Therefore, a reproducible and effective *in vitro* system for plant regeneration is crucial for genetic improvement of ‘Egusi’. A limited number of studies have been documented for *in vitro* plant regeneration systems through organogenesis. Ntui et al. (2009) reported the establishment of efficient plant regeneration system of three ‘Egusi’ genotypes, ‘‘Ejagham’’, ‘‘Sewere’’ and ‘‘Barablackedge’’ using cotyledons of *in vitro*-germinated seedlings as explants. High regeneration efficiencies were observed on Murashige and Skoog’s (MS) medium supplemented with BAP when the cotyledons were cut into halves (Ntui et al., 2009). Thangavel et al. (2019) reported a system for large scale multiple shoot bud regeneration from nodal explants of ‘Egusi’ (*Citrullus colocynthis*). They observed high frequency shoot regeneration when nodal explants were cultured in MS medium supplemented with benzyl amino purine BAP (1.0 mg/l), kinetin (0.5 mg/l), gibberellic acid (1.5 mg/l). Idehen et al. (2012) attempted regeneration of ‘Egusi’ using cotyledons as explant. Though the seeds germinated and produced cotyledons, regeneration frequency was very low. Somatic embryogenesis method is practicable for plant propagation and a resource of genetic transformation. There is no study documenting regeneration of ‘Egusi’ through somatic embryogenesis. Production of embryos from cell suspension culture using cotyledons, nodes or leaf-derived callus could increase the regeneration efficiency of ‘Egusi’ melon even in the recalcitrant genotypes.

Recombinant DNA technology is often used to insert genes exhibiting important characteristics such as plant architecture, nutrient enrichment, high yield, excellent fruit quality, resistance to abiotic and biotic stresses, which will aid in the creation of new genotypes without substantially changing the desired phenotypic traits and genetic makeup of the cultivars. Developing genetically modified organism requires a highly efficient system for transformation. *Agrobacterium*-mediated transformation or microprojectile bombardment have been used to deliver transgenes into different melon species (Sultana and Rahman, 2013). To date, only one group has successfully reported the transformation of ‘Egusi’. Ntui et al. (2010a) generated transgenic plantlets from *in vitro* cotyledons by *Agrobacterium*-mediated genetic transformation using the GUS reporter gene and NPTII gene for selection in media containing kanamycin. Using the same principles, the group reported the production of ‘Egusi’ exhibiting resistance to Fusarium wilt and Alternaria leaf spot, some of the most devastating fungal diseases of ‘Egusi’, by overexpression of *Wasabi defensin* gene (Ntui et al., 2010b). *Wasabi defensin is* an anti-microbial peptide obtained from Wasabi (*Wasabia japonica*), a Japanese horseradish widely used in Japanese cuisine and as wrapping material to protect food from putrefaction (Saitoh et al., 2001). Defensins are low-molecular-weight (5 kDa) proteins present in the seeds, stems, roots, and leaves of many plant species that are toxic to bacteria, fungus, and yeast *in vitro*. It has been demonstrated that defensins permeabilize fungal membranes, which stops fungal growth and development (Thevissen et al., 1999).

The limited number of studies on plant regeneration and transformation of ‘Egusi’ points to the fact that ‘Egusi’ is still an orphan crop whose genetic and genomic resources have not been exploited to fast track its improvement and make its production profitable. The implementation of breeding programs that take advantage of these regeneration and transformation protocols would make rapid improvement of this crop possible. More efforts should be geared toward developing regeneration and transformation protocols for other ‘Egusi’ genotypes to facilitate their improvement.

Aside the mainstream biotechnological intervention via recombinant DNA technology, the possibilities of introducing a wider genetic variability in ‘Egusi’ melon via strategies like mutation breeding and the use of anti-mitotic agents need further exploration. During cell division, inhibiting the growth of spindle fibers using chemicals or herbicides could result in cells with doubled amount of genetic material. Plant microtubules can thus be targeted in applied plant breeding for the generation of haploid plants or the induction of polyploid offspring (Motta and Schnittger, 2021). In the curcubit plant - *Cucumis melo*, Cho et al. (2021) used oryzalin treatment to induce polyploidy in melon plants with 21% efficiency. The generated polyploids showed enhanced morphological characteristics. For ‘Egusi’ melon, an effort at ploidy manipulation was carried out by Brisibe et al. (2011) who demonstrated that soaking the seeds in solutions of the anti-mitotic agents - colchicine and oryzalin produced polyploid mutants with superior morphological and yield-related traits. However, despite this important effort, further research needs to be accelerated especially at *in vitro* ploidy manipulation given that a protocol for regenerating some genotypes of the plant *in vitro* is now available.

### 5.2 CRISPR/Cas9 strategy to develop improved ‘Egusi’ genotypes

Recently, the discipline of gene editing, which includes technologies that allow for precise modifications to an organism’s DNA, has made great strides. These technologies enable researchers to precisely add, remove, or alter genetic material at certain genomic regions. Several tools including mega nucleases, Zinc finger nucleases (ZFNs), TAL effector proteins (TALENs) and RNA-guided nucleases (RGENs) or CRISPR (clustered regularly interspaced short palindromic repeats)/Cas9 have been developed to target genome editing in plants and other organisms (Tripathi et al., 2024; Ntui et al., 2023). All these methods are based on the formation of double stranded breaks (DSBs) at specific loci and triggering DNA repair mechanism either through the non-homologous end joining (NHEJ) or homology-directed repair (HDR). The NHEJ pathway is an error-prone mechanism that leads to random insertions, deletions and substitutions (indels) at the cleavage sites, resulting in frameshift mutations and targeted gene knockouts. Conversely, the HDR process uses a homologous DNA repair template to allow for precise genomic modifications, such as gene knock-in, gene replacement, and insertion of foreign genes or DNA sequences. Depending on the repair mechanism, three types of editing outcomes namely: site-directed nuclease 1 (SDN1), SDN2 and SDN3 can be achieved. SDN1 results when NHEJ makes random changes in the gene, altering gene function or causing gene silencing or knockout. SDN2 employs a repair template that corresponds with the DSB, resulting in targeted indels or nucleotide substitution by HDR. SDN3 uses a longer repair template than homologous sequences to mend the DSB, which makes it easier for foreign genes to be inserted specifically (Kim and Kim, 2019).

Among the various gene editing tools, CRISPR/Cas has become a mainstay of genetic manipulation because of its efficiency, stability, ease of design, speed, cost-effectiveness, precision and ability to edit several genes at once. The CRISPR system is modelled after the type II CRISPR immune system found in bacteria, which protects against plasmids and/or DNA viruses. Two components comprise the CRISPR/Cas9 molecular immunity system: a synthetic single guide RNA (sgRNA) and the Cas9 endonuclease of *Streptococcus pyogenes.* A target sequence complementary to the 20 nucleotides preceding the protospacer adjacent motif (PAM) (NGG or NAG), which is required for Cas9 activity, guides the Cas protein to bind to the target sequence and starts editing 3 or 4 base pairs upstream of the PAM sequence. Generally, Cas9 has more affinity to NGG than NAG. Through the suppression of either endogenous or foreign genes, gRNAs may precisely direct the endonuclease Cas9 to cleave a target location and cause gene disruptions. By multiplexing, two or more genes can be edited simultaneously by the CRISPR/Cas9 system (Tripathi et al., 2024).

In addition to Cas9, numerous Cas variations have been developed, including as Cas12a, Cas13, Cas14, CasX and OMEGA. Cas12a, commonly referred to as Cpf1, a type V, class 2 CRISPR contains the RuvC domain only. It possesses crRNA biogenesis RNase and single-strand Dnase activity and recognizes T-rich PAM, TTN/TTTN/TTTV (N = A/T/C/G; V = A/C/G). Using a single sequence array on the chosen gRNA, Cas12a may be utilized for multiplex gene modification. Cas13a, a class 2 type VI-A ribonuclease, can locate and cleave ssRNA molecules found in the phage genome. It can differentiate between RNA viruses and identify viruses with more accuracy than PCR (Tripathi et al., 2024).

Cas14 is a nuclease that can be programmed using RNA guidance. It stands out for its ability to identify its target DNA independent of the protospacer adjacent motif (PAM), setting it apart as the sole DNA-targeted Cas that does not rely on PAM. Cas14 creates an R-loop formation by cleaving ssDNA without discrimination, utilizing short sgRNA, and identifying ssDNA that complements the sgRNA’s recognition arm (Wu et al., 2022; Zhou et al., 2023). Compared to Cas12a, Cas14 has a high level of specificity and sensitivity in detecting single-nucleotide polymorphisms (SNPs), making it useful for pathogen differentiation and genotyping. Research has delved into the indirect detection of non-nucleic acid targets using Cas14, enabling the precise identification of antibiotics with low nanomolar sensitivity. The use of metal isotopes in LC-MS, though, made the process more complex. Wu et al. (2022) developed a CRISPR/Cas14-based aptasensor with nanomaterials to enhance fluorescence signals, enabling highly sensitive detection of microcystin-LR for environmental monitoring. Using complementary DNA to interfere with the aptamer’s binding ability could lead to a decrease in the signal produced during target identification. Moreover, the capability of CRISPR/Cas14 for aptasensing has not yet been explored. Therefore, attempting to develop a Cas14-based biosensor for versatile aptasensing that is inexpensive, fast, and direct is worth it.

CasX was developed by analyzing bacteria from groundwater using metagenomics, and was characterized as an RNA-directed DNA cutting enzyme. By identifying a 5’-TTCN PAM and providing sgRNA (a crRNA-tracrRNA linked together), it has the ability to enhance plasmid interference in *E. coli* (Liu et al., 2019). The only resemblance to other recognized Cas endonucleases is a RuvC domain located at the end of the C-terminus. The traits of CasX are comparable to those of type V Cas12, but CasX is smaller than the reported size of Cas12 at around 980 amino acids, while Cas12 has approximately 1200 amino acids. Cas12 shows PAM-independent non-specific ssDNA trans-cleavage activity when bound to crRNA-guide-complementary ssDNA. CasX has lower trans-ssDNA cleavage activity compared to Cas12a and Cas12b. Yang and Patel (2019) explored the option of utilizing CasX to alter genes in human and *E. coli* cells.

A new group of RNA-guided endonucleases termed OMEGA, was recently discovered, which has a common core domain with the CRISPR-Cas12 family. The OMEGA system, comprising of transposable elements TnpB and Fanzor effectors, contains a CRISPR-Cas12-like domain (RuvC) which acts as an RNA-guided endonuclease (Karvelis et al., 2021). TnpB aids the TnpA module in transposing a specific locus by using uRNA that matches the target DNA sequence. The RNA guidance enables the reprogramming of DNA targeting, a technique commonly used in genome editing. The CRISPR-Cas12 system found in prokaryotes is thought to have evolved from TnpB by adding additional domains, as the original TnpBs only contains a basic domain that carries out the function of the CRISPR-Cas12 family. The characteristics of how TnpB targets DNA have been documented, from the earliest classified ISDra2 TnpB, K, and racemifer TnpB types to the latest database-screened ISDge10, ISAam1, and ISYmu1 (Altae-Tran et al., 2021; Sasnauskas et al., 2023; Xiang et al., 2023; Badon et al., 2024).

TnpB may also be the ancestor of the eukaryotic transposon-encoded Fanzor (Fz) proteins. Fanzor effectors are primarily present in plants, fungi, protists, arthropods, and eukaryotic viruses. On a molecular scale, they show significant similarities to the TnpB system. Fanzor is primarily split into two categories: Fanzor 1 and Fanzor 2. It has been noticed that both types recognize target-adjacent motif (TAM) sequence and uRNA that is complementary to target DNA, similar to TnpB, in order to create RNA-DNA heteroduplexes on target DNA. Similar to TnpB and Fanzor endonucleases, IscB also identifies TAM and has a modest size of 496 amino acids (OgeuIscB). Nevertheless, it presents similar capabilities, nucleic acid binding, and domain structure (RuvC, BH, and HNH domains) (Badon et al., 2024).

Prime and Base Editing are two additional editing programs developed using CRISPR technology. These editing techniques utilize dCas9, a altered form of Cas9. In order to create a base editor enabling base substitution at the level of single nucleotides without requiring a DNA donor template, a DNA deaminase is linked with dCas9. The effectors enable substituting C:G-to-T:A or A:T-to-G:C based on the type of DNA deaminase, while the RNA-guided CRISPR system identifies the specific location in the genome to be modified. Prime editing facilitates the exchange of DNA base pairs, small insertions, and slight deletions through a mechanism akin to traditional CRISPR/Cas systems (Chen et al., 2021; Matsoukas, 2020). On the other hand, primer editing eliminates the need for a donor template and avoids causing a double-strand break (DSB), while addressing off-target impacts and correcting frame shifts due to indels. The only way to modify the genome is with a fusion protein consisting of a longer gRNA called pegRNA and Cas9 H840A nickase attached to a modified reverse transcriptase (RT) enzyme. Prime editing shows potential as a supplement to existing CRISPR editing methods by enabling precise and focused alterations to DNA, despite the fact that its specific workings are still uncertain. Nevertheless, primary editing is a possible method that may be utilized to alter the genetic makeup of crops. Prime and base editing fall under the category of SDN1 due to their independence from a DNA donor template. This implies that edited crops could potentially be handled the same way as non-transgenic crops and might not have to follow strict biosafety rules.

A more recent advances in CRISPR is its use to activate or repress the expression of genes (Park et al., 2017). Introduction of mutations in the HNH (H840A) and RuvC (D10A) domains eliminates the endonuclease activity of Cas9 protein but retains the binding activity (Qi et al., 2013). When linked to transcriptional effector domains, the nuclease-dead Cas9 (dCas9) can be re-engineered to function as a transcriptional regulator (Moradpour and Abdulah, 2020). The development of inducible CRISPR/Cas9 transcriptional activator (CRISPRa) or repressor (CRISPRi) techniques offers tremendous promise for studying the effects of upregulating or silencing genes. These activator/repressor techniques which rely on the dCas9 variants linked to transcriptional activator/repressor domains, enables Cas9 to be utilized as a tool to modify transcription activity (Di Maria et al., 2020).

Another CRISPR/Cas9 system termed Cas-Clover was recently developed. Cas-CLOVER is a system that utilizes two guides to induce double-strand breaks upon binding with the nuclease Clo051 (Madison et al., 2022). The fusion protein contains a deactivated Cas9 (dCas9) protein linked to the Clo051 endonuclease as a binding protein at the target location in the genome of an organism. Unlike CRISPR, which uses only one guide RNA (gRNA), the Cas-CLOVER endonuclease system employs two gRNAs along with the Clo051 nuclease activity, which relies on the dimerization of subunits linked to each gRNA. The Cas-CLOVER genome editing system achieves high precision by utilizing two gRNAs, where Clo051 induces a double stranded break only when both gRNAs guide it to the correct target site in the plant genome. Clo051 displays dual gRNA localization, which occurs within an ideal spacer length ranging from 11 to 31 nucleotides, allowing for design flexibility. This method enables a more precise DNA cleavage with minimal risk of off-target effects, leading to longer, sticky overhangs instead of smaller blunt deletions seen with CRISPR/Cas9. The Cas-CLOVER gene editing technique is groundbreaking, extremely accurate, and off-target genetic changes are frequently difficult to detect (Madison et al., 2022).

Numerous attempts have been made to apply CRISPR in order to incorporate beneficial traits into various crops such as banana, maize, rice, soybeans, tomatoes, and wheat. In melon, not much has been done on genome editing. However, there are a few studies documenting the application of CRISPR/Cas9, this is mostly because melon genetic transformation is still a major problem. CRISPR/Cas9 system has been applied in melon to knockout *phytoene desaturase* (*PDS*) gene. PDS is a gene in the carotene biosynthetic pathway whose mutation causes photobleaching or albino phenotype. It is often used as a marker to establish genome editing in plants. Tian et al. (2017) produced albino watermelon plants by editing the *PDS* gene. They obtained a mutation efficiency of 100%, using a CRISPR/Cas9 construct containing two gRNAs. Similarly, the melon genotype *Cucumis melo* was edited by targeting the *PDS* gene (Hooghvorst et al., 2019). The authors produced a CRISPR/Cas9 system with two gRNAs, targeting exons 1 and 2 and delivered first into protoplasts by polyethylene glycol (PEG) transfection, and into the cotyledons by *Agrobacterium*-mediated transformation. Although the transformation efficiency was low, they recovered albino plants with a mutation efficiency of 42–45% after Sanger analysis.

Melon necrotic spot virus (MNSV) is a potyvirus which induces necrotic spots on leaves, Stems and fruits (Nonaka and Ezura, 2024). The recessive nsv resistance gene identified by Coudriet et al. (1981), encodes Cm-elF4E, which plays a role in translation initiation factors. One amino acid residue at 228Leu on Cm-elF4E exhibited susceptibility to all MNSV strains except MNSV-264 (Nieto et al., 2006). Using the CRISPR/Cas9-mediated cytosine base editor, Shirazi Parsa et al. (2023) introduced two kinds of mutations on Cm-elF4E, Cm-elF4E (C322T/C323G) with a stop codon and Cm-elF4E (C322T/C323T, P108L). Although the plants were not tested against MNSV, knocking out *eIF4E/iso4E* genes is a promising strategy for developing potyvirus-resistant plants and has been tried in several plant species (Tripathi et al., 2020).

In attempt to develop Papaya ringspot virus (PRSV) resistance in Melon, PRSV resistance candidate gene (Prv) was mutated using CRISPR/Cas9. *Prv* knockout mutants showed susceptibility to PRSV suggesting that Prv is a PRSV resistance gene (Nizan et al., 2023).

Ethylene plays a role in regulating the shelf-life of fruits and is one of the major promoters of fruit ripening. Prolonging the shelf-life of fruits decreases food waste, thus potentially enhancing food security. Depending on the involvement of ethylene during ripening, fruits have been divided into either climacteric (ethylene-dependent), non-climacteric (ethylene independent) (Giordano et al., 2022). Melon employs two types of ripening systems: climacteric and non-climacteric ripening, depending on the horticultural subgroups (Nonaka and Ezura, 2024). Repressor of silencing 1 (*ROS1*), constitutive triple response 1 (*CTR1*) and non-ripening (*NOR*) genes are reported to be associated with fruit ripening. ROS1 is required for normal fruit ripening, by the activation of ripening-induced genes and the repression of several ripening-repressed genes (Lang et al., 2017). CTR1, a serine/threonine kinase is a negative regulator of the ethylene signal transduction pathway (Kieber et al., 1993). NOR is related to ethylene signaling partway. Knockout of ROS1 and CTR1(CmCTR1-like, MELO3C024518) independently in climacteric melon by CRISPR/Cas9, accelerated fruit ripening in edited events compared to the wild-type (Giordano et al., 2022). Subsequently, knockout of CmNOR using the CRISPR/Cas9 system delays ripening in the climacteric melon (Liu et al., 2022). The enzyme 1-aminocyclopropane-1-carboxylic acid oxidase (ACO) is the final step of the ethylene production pathway (Nonaka et al., 2023). Modification of CmACO1 in *Cucumis melo* by CRISPR/Cas9 reduced ethylene production up to one-tenth that of the wild type in fruits stored at room temperature for 14 days, the pericarp color remained green, and the fruits had higher fruit firmness (Nonaka et al., 2023).

CRISPR/Cas9 has been demonstrated in watermelon to create herbicide resistant genotype by targeting acetolactate synthase (ALS) (Tian et al., 2018). ALS is the key enzyme for biosynthesis of branched-chain amino acids, valine, leucine and isoleucine, in plants. In many plant species, single point mutations at multiple conserved locations of the ALS gene have been shown to provide a significant degree of herbicide resistance (Yu and Powles, 2014). By targeting the ALS gene using base editing, Tian et al. (2018) successfully produced edited watermelon exhibiting tolerance to the herbicide tribenuron herbicide. Sequence analysis revealed 45 out of 199 transgenic plants had C to T mutations resulting in amino acid change. One advantage of targeting ALS is the production of foreign DNA-free edited plants which could be achieved by simultaneously editing ALS and a gene of interest. The main limitation of this method is that in the absence of selection several plants have to be screened to identify potential transgene-free edits. However, this approach would be useful for crops that are vegetatively propagated.

‘Egusi’ genomes can be edited in a similar way in a targeted manner to produce new varieties. We are currently working on developing genome editing platform for ‘Egusi’ by targeting *PDS* gene. The established protocol will pave the way for genome editing of ‘Egusi’ by modifying genes associated with disease resistance, fruit, and seed quality. Some of these genes are summarized in **Table 4**.

**Table 4:** Potential genes that could be targeted in ‘Egusi’ to develop resistance to diseases by genome editing.

Gene	Plant	Type of pathogen	Target pathogen	Mode of action	References
*CmVPS41*	Cucumber	Virus	Cucumber mosaic virus	RNAi restricts viral movement by altering cellular localization	Pascual et al., 2019; Real et al., 2023
***Wmv***	Watermelon	Virus	Watermelon mosaic virus	Not elucidated	Pérez-de-Castro et al., 2019
***NBL***	Zucchini	Virus	Zucchini yellow mosaic virus	Coiled coil-nucleotide binding site of the leucine-rich repeat (CC-NB-LRR)	Adler-Berke et al., 2021
***Prv***	Papaya	Virus	Papaya ringspot virus	Encodes a protein similar to Fom-1, involved in resistance to Fusarium oxysporum	Nizan et al., 2023
***Cm-elF4E***	Melon	Virus	Melon necrotic spot virus	Involved in translation initiation; specific mutations confer resistance	Shirazi Parsa et al., 2023
***eIF4E***	Cucumber	Virus	Cucumber Vein Yellowing Virus (Ipomovirus), Zucchini Yellow Mosaic Virus (ZYMV), Papaya Ringspot Virus-W (PRSV-W)	Introduce small deletions in targeted exons	Chandrasekaran et al., 2016
***Vat***	Melon	Virus via insect	Aphis gossypii	Provides resistance to aphid-mediated viral transmission	Chovelon et al., 2021
***MELO3C004311***	Melon	Fungi	Podosphaera xanthii, Golovinomyces cichoracearum	Powdery mildew resistance	López-Martín et al., 2022
***MELO3C010403***	Melon	Fungi	Gummy Stem Blight (GSB)	New candidate gene for resistance	Ma et al., 2023
***MELO03C012987***	Melon	Fungi	Gummy Stem Blight (GSB)	Dominant gene associated with GSB resistance	Hu et al., 2018
***Clpsk1***	Melon	Fungi	*Fusarium oxysporum* (FON)	Targeted mutation and loss-of function	Zhang et al., 2020
***Fom-1/Fom-2***	Melon	Fungi	*Fusarium oxysporum*	Targeted editing to improve resistance to multiple races of Fusarium oxysporum	Liu et al., 2022
***QTLs***	Melon	Fungi	Downy Mildew (DM)	Identified major QTLs including qPcub-10.1 and qPcub-8.2	Zhang et al., 2023
***DIPM-1 DIPM-2 DIPM-4***	Apple	Bacteria	Erwinia amylovora	Gene disruption	Malnoy et al., 2016
***MusaDMR6, MusaENOD3***	Banana	Bacteria	*Xanthomonas* *campestris* pv. *musacearum*	Gene disruption	Tripathi et al., 2021; Ntui et al., 2024
***citri (Xcc) CsLOB***	Citrus	Bacteria	Xanthomonas citri (Xcc)	Gene disruption	Jia et al., 2016
***OsSWEET13***	Rice	Bacteria	*Xanthomonas oryzae*	Gene disruption	Zhou et al., 2015
***OsPi21, OsBsr-d1, and OsXa5***	Rice	Bacteria	*Xanthomonas oryzae*	Gene disruption	Tao et al., 2021
***OsPi21 and OsXa13***	Rice	Bacteria	Xanthomonas oryzae	Gene disruption	Li et al., 2019
***SlJAZ2***	Tomato	Bacteria	*Pseudomonas syringae pv. toma*	Gene disruption	Ortigosa et al., 2019
***SlDMR6-1 and SlDMR6-2***	Tomato	Bacteria	*P. syringae pv. tomato, X. gardneri, and Xanthomonas perforans*	Gene disruption leading to loss of function	Thomazella et al., 2021

## 6 Challenges in developing improved ‘Egusi’ through genetic modification and genome editing

**Figure 5:**
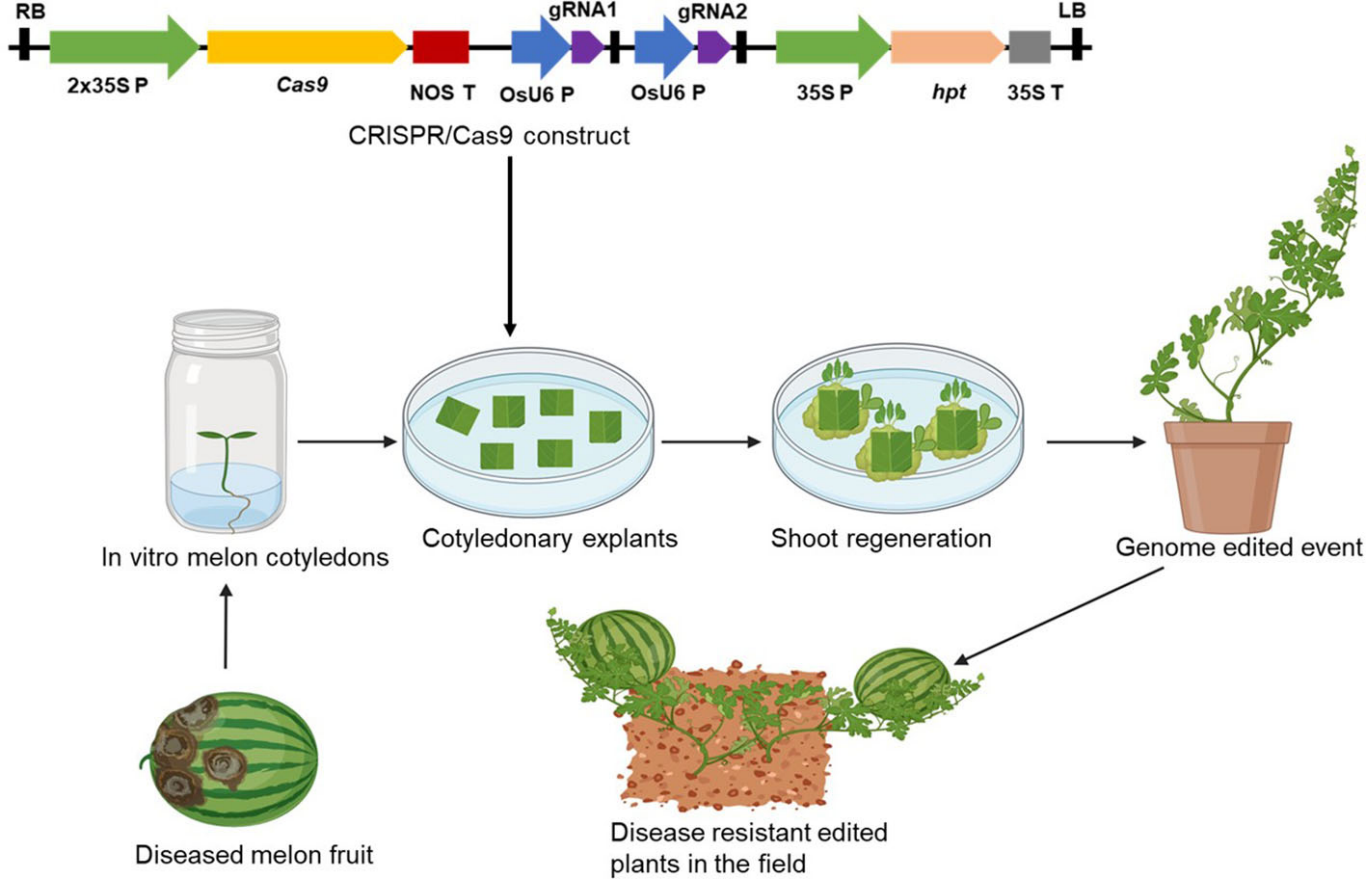
Process of producing disease resistant ‘Egusi’ melon genotype using CRISPR/Cas9 genome editing technique. The CRISPR/Cas9 construct is integrated into the cotyledonary explants and disease resistant genome edited plants are generated through tissue culture.

The biggest challenge in the application of genetic modification and CRISPR/Cas9 technology in ‘Egusi’ is the lack of efficient regeneration and genetic transformation protocol and regeneration of complete plants from transformed cells for many genotypes. Although plant regeneration and genetic transformation protocol by *Agrobacterium* using cotyledons as explants have been documented for some ‘Egusi’ genotypes, genetic transformation is cultivar-dependent. Moreover, melon genetic transformation is still a major challenge. Using genotypes whose transformation and regeneration protocols are well established, would facilitate the production of transgenic and genome edited events (**Figure 5**). ‘Egusi’ genotypes which are recalcitrant to transformation could be edited by delivering the CRISPR/Cas machinery directly to the genotypes by crossing these genotypes with a stably transformed cultivar harboring a CRISPR/Cas construct, allowing the continued activity of CRISPR/Cas to generate new mutated alleles (Hernandes-Lopes et al., 2023). Alternative strategies that could bypass genetic transformation should be adopted to deliver CRISPR/Cas machinery into ‘Egusi’ cells. Methods such as agroinfiltration or in planta transformation or the use of viral vector-based platforms for rapid and efficient delivery of CRISPR/Cas9 constructs could increase the chances of editing ‘Egusi’ genotypes that are recalcitrant to genetic transformation. Also, techniques such as mobile RNA, can be used to achieve genome editing in recalcitrant plants by grafting a recalcitrant shoot on a transgenic donor root plant without the need for tissue culture (Yang et al., 2023). For example, a transgenic genome edited root of ‘Egusi’ genotype which is amendable to tissue culture can be produced. On grafting the root with a recalcitrant shoot, the Cas9 and the gRNA transcripts will be transported from the transgenic rootstock to the shoot of the recalcitrant ‘Egusi’ plant, where they create mutations in the shoots without integrating into the genome. The resultant seeds and shoots are genome-edited lines devoid of foreign DNA.

In order to utilize genome editing as a breeding tool in ‘Egusi’, obtaining genome and genetic information is essential. Melon’s genetic analysis has been conducted and the size of its genome was estimated to be around 454Mb using nuclear DNA content measurements (Arumuganathan and Earle, 1991; Garcia-Mas et al., 2012). Whole genome sequence for *‘Egusi’, collected from Nigeria, and referred to as Citrullus mucosospermus, in the cucurbit genomics is available* at Melonomics v4.0 (Ruggieri et al., 2018, https://www.melonomics.net) and CuGenDBv2 (Yu et al., 2023, http://cucurbitgenomics.org/v2/), which could be used as a reference genome for ‘Egusi’ genome editing.

Selecting a good target gene and region for editing may have influence on the desired phenotype. When choosing target genes and regions for genome editing, it is essential to consider not only gene function but also the location and timing of gene expression. There is a publicly accessible gene expression database that reveals the site, timing, and intensity of gene expression (Yano et al., 2018; Yano et al., 2020). While coding sequences are typically the preferred targets for gene editing, other genetic elements, such as regulatory sequences, can also be focused on to adjust spatial and temporal gene expression patterns. For example, genes that are crucial in the process of plant domestication exhibit more consistent expression patterns in cultivated plants compared to their wild counterparts, indicating that particular cis-regulatory elements (CREs) were chosen during the domestication process (Lemmon et al., 2014; Swinnen et al., 2016). So, gene editing can focus on regulatory sequences to adjust gene expression levels and tissue preferences for the development of various subtle traits.

There is concern on whether genome edited products should be treated as GMOs. Many countries including Nigeria and Ghana, which are among the highest producers of ‘Egusi’ have developed guidelines for genome editing. In these countries, genome editing products containing no foreign genes (mostly SDN1 and to some extent, SDN2) are not regulated as GMO and as such will be treated as products generated by conventional breeding. ‘Egusi’ is a diploid, therefore, transgenes can easily be segregated out by crossing even if the edits were generated by plasmid delivery and *Agrobacterium*, producing foreign DNA-free edited plants. Hence, genome edited ‘Egusi’ will not be treated as GMOs by regulatory bodies and will not go through the stringent biosafety regulations before being released in the case of a product, which will increase its commercialization and acceptability.

Often when a new technology is developed, the public reacts with fear of unknowns, especially those related to food. The introduction of genome editing, like all new technologies, has sparked worldwide legal and ethical discussions, which vary depending on the type of gene editing involved. One of the key ethical issues surrounding the use of gene editing is religion. A lot of individuals hold ethical and religious reservations regarding the utilization of gene editing products. Editing the genes of organisms is seen by many as either tampering with God’s creation or taking on the role of God. This might cause issues with the acceptance of genome edited ‘Egusi’ products especially in highly religious countries like Nigeria.

Another main concern is off-target effects (editing wrong gene or genes) and mosaicism leading to undesired traits. Safety in gene editing has been a crucial concern. Doubts exist about the safety of gene editing, particularly when applied to gene drives, as the full implications of off-target effects in gene editing remain unclear. Although there are concerns about off-target effects, this is taken care of during guide design, where only gRNAs with no off-target effects are selected for editing and many CRISPR software are able to detect guides with potential off-target effects.

Another key concern regarding gene editing revolves around establishing regulatory frameworks for the technology and determining whether genetically edited crops should be classified as GMOs or not. While some countries have clarified their rules on the release of genetically modified crops, others like the European Union (EU) and New Zealand have strict regulations, causing differences in national regulatory approaches. When developing policies for gene editing in plant breeding, considerations should also include farmer’s rights and public acceptance (Idris et al., 2023). Just like with most new technologies, there is worry about Intellectual Property Rights (IPR). Gene-edited items will be patented, giving owners, usually agri-food companies, exclusive rights to the gene-editing process’ results, essentially creating a monopoly over them. This is likely to exacerbate the existing moral dilemma of power imbalances in agriculture between big corporations and small, traditional farmers.

## 7 Conclusion

‘Egusi’ is one of the most important cucurbits cultivated in Africa. There is a lack of improvement programs most probably because of neglect in terms of research. At a time when the world is dealing with climate change and a growing population, it is crucial to utilize various breeding and biotechnological methods to produce ‘Egusi’ that can withstand the effects of climate change. This review has attempted to present current gaps in ‘Egusi’ breeding and emphasized the importance of the applications of genomics, transcriptomics, proteomics, and genome editing for the improvement and sustainability of ‘Egusi’ melon.

## References

[B1] Abushamleh N. H. El-Keblawy A. Mosa K. A. Soliman S. S. Tsombou F. M. (2022). Different Traits Affect Salinity and Drought Tolerance during Germination of Citrullus colocynthis, a Potential Cash Crop in Arid Lands. Seeds 1, 244–259. doi: 10.3390/seeds1040021

[B2] Achigan-Dako E. G. Fagbemissi R. Avohou H. T. Vodouhe R. S. Coulibaly O. Ahanchede A. (2008). Importance and practices of egusi crops (Citrullus lanatus (Thunb.) matsum. & nakai, cucumeropsis mannii naudin and lagenaria siceraria (Molina) standl. cv. ‘Aklamkpa’) in sociolinguistic areas in benin. Biotechnol. Agron. Soc Environ. 12 (4), 393–403.

[B3] Adler-Berke N. Goldenberg Y. Brotman Y. Kovalski I. Gal-On A. Doniger T.. (2021). The melon *Zym* locus conferring resistance to ZYMV: High resolution mapping and candidate gene identification. Agron. 11 (12), 2427. doi: 10.3390/agronomy11122427

[B4] Akashi K. Yoshida K. Kuwano M. Kajikawa M. Yoshimura K. Hoshiyasu S.. (2011). Dynamic changes in the leaf proteome of a C3 xerophyte, Citrullus lanatus (wild watermelon), in response to water deficit. Planta 233, 947–960. doi: 10.1007/s00425-010-1341-4 21259065

[B5] Akubor P. I. Obasi B. C. (2019). Evaluation of the quality of a beverage prepared from toasted melon seeds. South Asian J. Food Technol. Environ. 5, 763–770. doi: 10.46370/sajfte.2019.v05i01.02

[B6] Akubor P. I. Ogbadu R. I. (2003). Effects of processing methods on the quality and acceptability of melon milk. Plant Foods Hum. Nutr. 58, 1–6. doi: 10.1023/a:1024063105507 12859008

[B7] Al-Shuaibi B. K. Kazerooni E. A. Hussain S. Velazhahan R. Al-Sadi A. M. (2023). Plant-Disease-Suppressive and growth-promoting activities of endophytic and rhizobacterial isolates associated with citrullus colocynthis. Pathogens 12 (11), 1275. doi: 10.3390/pathogens12111275 38003740 PMC10674396

[B8] Alsaiari R. A. Musa E. M. Rizk M. A. (2023). Biodiesel production from date seed oil using hydroxyapatite derived catalyst from waste camel bone. Heliyon 9, e15606. doi: 10.1016/j.heliyon.2023.e15606 37144194 PMC10151356

[B9] Altae-Tran H. Kannan S. Demircioglu F. E. Oshiro R. Nety S. P. McKay L. J.. (2021). The widespread IS200/IS605 transposon family encodes diverse programmable RNA-guided endonucleases. Science 374, 57–65. doi: 10.1126/science.abj6856 34591643 PMC8929163

[B10] Amali E. P. Kortse P. A. Vange T. (2013). The quality of ‘egusi’ melon [(Citrullus lanatus thunb.) matsum and nakai] seeds derived from fruits harvested at different growth stages and at different positions on the mother plant. Int. J. Sci. Res. Pub. 3 (4), 1–7.

[B11] Amanullah S. Liu S. Gao P. Zhu Z. Zhu Q. Fan C.. (2018). QTL mapping for melon (*Cucumis melo* l.) fruit traits by assembling and utilization of novel SNPs based CAPS markers. Sci. Hortic. 236, 18–29. doi: 10.1016/j.scienta.2018.02.041

[B12] Arthur W. Ofori J. Addo P. Amey N. Kortei N. K. Akonor P. T. (2020). Chemical, Microbial Quality, and Risk Assessment due to Toxic Metal Contamination of ‘Egusi’ (*Citrullus colocynthis* L.) Powder Sold in Ghanaian Markets. Int. J. Food Sci. 2020, 8862404. doi: 10.1155/2020/8862404 33381541 PMC7748910

[B13] Arumuganathan K. Earle E. D. (1991). Nuclear DNA content of some important plant species. Plant Mol. Biol. Rep. 9, 208–218. doi: 10.1007/bf02672016

[B14] Azuaga I. C. Igbum G. O. Kyenge B. A. (2018). Extraction and Characterization of Three Tropical Seedoils: Telfairia occidentalis, Hura crepitans and Cucumeropsis mannii. Chem. Res. J. 3, 1–8.

[B15] Beaulieu J. C. (2005). Within-season volatile and quality differences in stored fresh-cut cantaloupe cultivars. J. Agric. Food Chem. 53 (22), 8679–8687. doi: 10.1021/jf050241w 16248571

[B16] Badon I. S. Oh Y. Kim H.-J. Lee S. H. (2024). Recent application of CRISPR-Cas12 and OMEGA system for genome editing. Mol. Ther. 32, 32–43. doi: 10.1016/j.ymthe.2023.11.013 37952084 PMC10787141

[B17] Baillo E. H. Kimotho R. N. Zhang Z. Xu P. (2019). Transcription factors associated with abiotic and biotic stress tolerance and their potential for crops improvement. Genes 10 (10), 771. doi: 10.3390/genes10100771 31575043 PMC6827364

[B18] Bello E. I. Makanju A. (2011). Performance evaluation of egunsi melon (*Citrullus colocynthis* L.) seeds oil biodiesel. J. Emer. Trends Eng. Appl. Sci. 2, 741–745.

[B19] Belouah I. Nazaret C. Pétriacq P. Prigent S. Bénard C. Mengin V.. (2019). Modeling protein destiny in developing fruit. Plant Physiol. 180, 1709–1724. doi: 10.1104/pp.19.00086 31015299 PMC6752906

[B20] Blum M. Waldner M. Olaya G. Cohen Y. Gisi U. Sierotzki H. (2011). Resistance mechanism to carboxylic acid amide fungicides in the cucurbit downy mildew pathogen pseudoperonospora cubensis. Pest Manag Sci. 67 (10), 1211–1214. doi: 10.1002/ps.2238 21780281

[B21] Brahma S. Nath B. Basumatary B. Das B. Saikia P. Patir K.. (2022). Biodiesel production from mixed oils: A sustainable approach towards industrial biofuel production. Chem. Eng. J. Adv. 10, 100284. doi: 10.1016/j.ceja.2022.100284

[B22] Brisibe E. A. Udensi O. Ntui V. O. Otu P. A. Chukwurah P. N. (2011). Sensitivity of some quantitative and yield characters of ‘Egusi’ melon (*Colocynthis citrullus* L.) to treatment with microtubule inhibitors. Afr. J. Plant Sci. 5, 759–766. doi: 10.5897/AJPS11.240

[B23] Buasri A. Chaiyut N. Loryuenyong V. Worawanitchaphong P. Trongyong S. (2013). Calcium oxide derived from waste shells of mussel, cockle, and scallop as the heterogeneous catalyst for biodiesel production. Sci. World J. 2013, 460923. doi: 10.1155/2013/460923 PMC3881677 24453854

[B24] Burkill H. M. (1985). The useful plants of west tropical Africa (Kew, UK: Royal Botanic Gardens), Vol. 1. 577.

[B25] Call A. D. Criswell A. D. Wehner T. C. Klosinska U. Kozik E. U. (2011). Screening cucumber for resistance to downy mildew caused by pseudoperonospora cubensis (Berk. and curt.) rostov. Crop Sci. 52, 577–592. doi: 10.2135/cropsci2011.06.0296

[B26] Chandrasekaran J. Brumin M. Wolf D. Leibman D. Klap C. Pearlsman M.. (2016). Development of broad virus resistance in non-transgenic cucumber using CRISPR/Cas9 technology. Mol. Plant Pathol. 17, 1140–1153. doi: 10.1111/mpp.12375 26808139 PMC6638350

[B27] Chen L. Park J. E. Paa P. Rajakumar P. D. Prekop H.-T. Chew Y. T.. (2021). Programmable C:G to G:C genome editing with CRISPR/Cas9-directed base excision repair proteins. Nat. Commun. 12, 1384. doi: 10.1038/s41467-021-21559-9 33654077 PMC7925527

[B28] Cheng H. Kong W. Tang T. Ren K. Zhang K. Wei H.. (2022). Identification of key gene networks controlling soluble sugar and organic acid metabolism during oriental melon fruit development by integrated analysis of metabolic and transcriptomic analyses. Front. Plant Sci. 13, 830517. doi: 10.3389/fpls.2022.830517 35646021 PMC9135470

[B29] Cho W.-Y. Deepo D. M. Islam M. M. Nam S.-C. Kim H.-Y. Han J.-S.. (2021). Induction of polyploidy in Cucumis melo ‘Chammel’ and evaluation of morphological and cytogenetic changes. Hortic. Sci. Technol. 39, 625–636. doi: 10.7235/hort.20210056

[B30] Chovelon V. Feriche-Linares R. Barreau G. Chadoeuf J. Callot C. Gautier V.. (2021). Building a cluster of NLR genes conferring resistance to pests and pathogens: the story of the *Vat* gene cluster in cucurbits. Hortic. Res. 8, 72. doi: 10.1038/s41438-021-00507-0 33790238 PMC8012345

[B31] Coudriet D. L. Kishaba A. N. Bohn G. W. (1981). Inheritance of resistance to Muskmelon necrotic spot virus in a melon aphid resistant breeding line of muskmelon. J. Am. Soc. Hortic. Sci. 106, 789–791. doi: 10.21273/JASHS.106.6.789

[B32] da Silva Castro L. Barañano A. G. Pinheiro C. J. G. Menini L. Pinheiro P. F. (2019). Biodiesel production from cotton oil using heterogeneous CaO catalysts from eggshells prepared at different calcination temperatures. Green Process Synth. 8, 235–244. doi: 10.1515/gps-2018-0076

[B33] Demirci S. Fuentes R. R. van Dooijeweert W. Aflitos S. Schijlen E. Hesselink T.. (2021). Chasing breeding footprints through structural variations in Cucumis melo and wild relatives. G3 (Bethesda) 11 (1), jkaa038. doi: 10.1093/g3journal/jkaa038 33561242 PMC8022733

[B34] Díaz A. Zarouri B. Fergany M. Eduardo I. Alvarez J. M. Picó B.. (2014). Mapping and introgression of QTL involved in fruit shape transgressive segregation into “piel de sapo” melon (cucumis melo l.) [corrected. PloS One 9, e104188. doi: 10.1371/journal.pone.0104188 25126852 PMC4134209

[B35] Di Maria V. Moindrot M. Ryde M. Bono A. Quintino L. Ledri M. (2020). Development and validation of CRISPR activator systems for overexpression of CB1 receptors in neurons. Front. Mol. Neurosci. 13. doi: 10.3389/fnmol.2020.00168 PMC7506083 33013319

[B36] Dogan M. Wang Z. Cerit M. Valenzuela-Antelo J. L. Dhakal S. Chu C.. (2023). QTL analysis of yield and end-use quality traits in Texas hard red winter wheat. Agronomy 13, 689. doi: 10.3390/agronomy13030689

[B37] Egwim E. C. Akanya H. O. Onuekwusi E. C. Ossamalu I. F. Mohammed J. M. (2015). Physicochemical and organoleptic properties of some selected foods fried with melon (*Citrullus lanatus*) seed oil. IOSR J. Biotechnol. Biochem. 1, 43–48.

[B38] Elnaggar A. Tsombou F. M. Hussain M. I. Almehdi A. M. Abideen Z. Wan Hong Yong J.. (2024). Citrullus colocynthis regulates photosynthetic and biochemical processes to develop stress resilience and sustain growth under sub-optimal temperatures. Plant Stress 12, 100502. doi: 10.1016/j.stress.2024.100502

[B39] Enujiugha V. N. Adeyemo M. B. Adisa A. M. (2023). Nutritional and safety implications of consuming melon seeds and impacts on international trade: A review. Food Humanity 1, 241–249. doi: 10.1016/j.foohum.2023.05.020

[B40] Essien E. A. Umoren S. A. Essien E. E. Udoh A. P. (2012). Preparation and evaluation of Cucumeropsis mannii Naud. seed oil metallic soaps as driers in gloss paint. J. Mater. Environ. Sci. 3, 477–484.

[B41] Etim A. O. Musonge P. Eloka-Eboka A. C. (2022). Process optimization of bio-alkaline catalysed transesterification of flax seed oil methyl ester. Sci. Afr. 16, e01275. doi: 10.1016/j.sciaf.2022.e01275 33431775

[B42] Fan M. Xu Y. Zhang H. Y. Ren H. Z. Kang G. B. Wang Y. J.. (2000). Identification of quantitative trait loci associated with fruit traits in watermelon [Citullus lanantus (Thanb) Mansf] and analysis of their genetic effects. Yi Chuan xue bao = Acta Genetica Sin. 27, 902–910. 11192435

[B43] FAO (2022). FAOSTAT Data on Crops: Melon seed (Food and Agricultural Organization of the United Nations) (Accessed June 16, 2024).

[B44] FAOSTAT. (2022). Data on yield of crops and area harvested. (Rome, Italy: Food and Agricultural Organization of the United Nations, FAO). Available online at: https://www.fao.org (Accessed June 11, 2024).

[B45] Flores F. El Yahyaoui F. de Billerbeck G. Romojaro F. Latche A. Bouzayen M.. (2002). Role of ethylene in the biosynthetic pathway of aliphatic ester aroma volatiles in charentais cantaloupe melons. J. Exp. Bot. 53, 201–206. doi: 10.1093/jexbot/53.367.201 11807123

[B46] Fokou E. Achu M. B. Kansci G. Ponka R. Fotso M. Tchiegang C.. (2009). Chemical properties of some Cucurbitaceae oils from Cameroon. Pakistan J. Nutr. 8, 1325–1334. doi: 10.3923/pjn.2009.1325.1334

[B47] Ford K. L. Cassin A. Bacic A. (2011). Quantitative proteomic analysis of wheat cultivars with differing drought stress tolerance. Front. Plant Sci. 2, 44. doi: 10.3389/fpls.2011.00044 22639595 PMC3355674

[B48] Gaide I. Makareviciene V. Sendzikiene E. Kazancev K. (2021). Natural rocks–heterogeneous catalysts for oil transesterification in biodiesel synthesis. Catalysts 11, 384. doi: 10.3390/catal11030384

[B49] Galpaz N. Gonda I. Shem-Tov D. Barad O. Tzuri G. Lev S.. (2018). Deciphering genetic factors that determine melon fruit-quality traits using RNA-Seq-based high-resolution QTL and eQTL mapping. Plant J.: For Cell Mol. Biol. 94, 169–191. doi: 10.1111/tpj.13838 29385635

[B50] Gao W. She F. Sun Y. Han B. Wang X. Xu G. (2023). Transcriptome analysis reveals the genes related to water-melon fruit expansion under low-light stress. Plants 12 (4), 935. doi: 10.3390/plants12040935 36840282 PMC9958833

[B51] Garcia-Mas J. Benjak A. Sanseverino W. Bourgeois M. Mir G. González V. M.. (2012). The genome of melon (Cucumis melo L.). Proc. Natl. Acad. Sci. U.S.A. 109, 11872–11877. doi: 10.1073/pnas.1205415109 22753475 PMC3406823

[B52] Gardner K. M. Brown P. Cooke T. F. Cann S. Costa F. Bustamante C.. (2014). Fast and cost-effective genetic mapping in apple using next-generation sequencing. G3 4, 1681–1687. doi: 10.1534/g3.114.011023 25031181 PMC4169160

[B53] Giordano A. Santo Domingo M. Quadrana L. Pujol M. Martín-Hernández A. M. Garcia-Mas J. (2022). CRISPR/Cas9 gene editing uncovers the roles of CONSTITUTIVE TRIPLE RE-SPONSE 1 and REPRESSOR OF SILENCING 1 in melon fruit ripening and epigenetic regulation. J. Exp. Bot. 73, 4022–4033. doi: 10.1093/jxb/erac148 35394503

[B54] Giwa S. Abdullah L. C. Adam N. M. (2010). Investigating “’Egusi’” (*Citrullus colocynthis* L.) seed oil as potential biodiesel feedstock. Energies 3, 607–618. doi: 10.3390/en3040607

[B55] Gómez G. Torres H. Pallás V. (2005). Identification of translocatable RNA-binding phloem proteins from melon, potential components of the long-distance RNA transport system. Plant J.: For Cell Mol. Biol. 41, 107–116. doi: 10.1111/j.1365-313x.2004.02278.x 15610353

[B56] Gonzalez-Ibeas D. Cañizares J. Aranda M. A. (2012). Microarray analysis shows that recessive resistance to Watermelon mosaic virus in melon is associated with the induction of defense response genes. Mol. Plant-Microbe Interact.: MPMI 25, 107–118. doi: 10.1094/mpmi-07-11-0193 21970693

[B57] Guo S. Zhao S. Sun H. Wang X. Wu S. Lin T.. (2019). Resequencing of 414 cultivated and wild watermelon accessions identifies selection for fruit quality traits. Nat. Genet. 51, 1616–1623. doi: 10.1038/s41588-019-0518-4 31676863

[B58] Hairuddin A. A. Tobib H. M. Zulkifli F. Noor M. M. (2019). The performance of a single-cylinder diesel engine fuelled with ‘Egusi’ based biodiesel. IOP Conf. Ser.: Mater. Sci. Eng. 469, 012045. doi: 10.1088/1757-899X/469/1/012045

[B59] Hangun-Balkir Y. (2016). Green biodiesel synthesis using waste shells as sustainable catalysts with *Camelina sativa* oil. J. Chem. 2016, 6715232. doi: 10.1155/2016/6715232

[B60] Hernandes-Lopes J. Yassitepe J. E. C. T. Koltun A. Pauwels L. Silva V. C. H. D. Dante R. A.. (2023). Genome editing in maize: Toward improving complex traits in a global crop. Genet. Mol. Biol. 46, e20220217. doi: 10.1590/1678-4685-GMB-2022-0217 36880696 PMC9990078

[B61] Hidayat A. Sakariel N. S. Tambunan L. A. (2022). Biodiesel synthesis from coconut oil using calcined scallop shell waste as the heterogeneous catalysts. Mater. Sci. Forum 1073, 155–160. doi: 10.4028/p-s9j02h

[B62] Hooghvorst I. López-Cristoffanini C. Nogués S. (2019). Efficient knockout of phytoene desaturase gene using CRISPR/Cas9 in melon. Sci. Rep. 9, 17077. doi: 10.1038/s41598-019-53710-4 31745156 PMC6863862

[B63] Hu Z. Deng G. Mou H. Xu Y. Chen L. Yang J.. (2018). A re-sequencing-based ultra-dense genetic map reveals a gummy stem blight resistance-associated gene in *Cucumis melo* . DNA Res. 25 (1), 1–10. doi: 10.1093/dnares/dsx033 28985339 PMC5824858

[B64] Idehen E. O. Kehinde O. B. Wang X. Popoola A. R. Akintobi D. C. (2012). Germination and I n-Vitro Regeneration in ‘‘Egusi’’ Melon, *Citrullus lanatus* (Thunb.) Matsum. and Nakai. Nigerian J. Biotechnol. 24, 35–40.

[B65] Idris S. H. Mat Jalaluddin N. S. Chang L. W. (2023). Ethical and legal implications of gene editing in plant breeding: a systematic literature review. J. Zhejiang Univ. Sci. B 24, 1093–1105. doi: 10.1631/jzus.b2200601 38057267 PMC10710910

[B66] Ilodibia C. V. Achebe U. A. Udeorah S. N. Okeke N. F. Ezeabara C. A. (2014). Growth and yield response of “ ‘Egusi’ Melon (*Citrullus lanatus* L.) to different nutrient sources in ultisol of South-Eastern Nigeria. Nigeria Agricul. J. 45(2), 21–6.

[B67] Jia H. Orbovic V. Jones J. B. Wang N. (2016). Modification of the PthA4 effector binding elements in Type I CsLOB1 promoter using Cas9/sgRNA to produce transgenic Duncan grapefruit alleviating Xcc1pthA4:dCsLOB1.3 infection. Plant Biotechnol. J. 14, 1291–1301. doi: 10.1111/pbi.12495 27071672 PMC11389130

[B68] Karvelis T. Druteika G. Bigelyte G. Budre K. Zedaveinyte R. Silanskas A.. (2021). Transposon-associated TnpB is a programmable RNA-guided DNA endonuclease. Nature 599, 692–696. doi: 10.1038/s41586-021-04058-1 34619744 PMC8612924

[B69] Khabiti M. Khorasheh F. Larimi A. (2021). Biodiesel production via transesterification of canola oil in the presence of Na–K doped CaO derived from calcined eggshell. Renewable Energy 163, 1626–1636. doi: 10.1016/j.renene.2020.10.039

[B70] Kieber J. J. Rothenberg M. Roman G. Feldmann K. A. Ecker J. R. (1993). CTR1, a negative regulator of the ethylene response pathway in Arabidopsis, encodes a member of the Raf family of protein kinases. Cell 72, 427–441. doi: 10.1016/0092-8674(93)90119-b 8431946

[B71] Kim J. Kim J. (2019). New era of precision plant breeding using genome editing. Plant Biotechnol. Rep. 13, 419–421. doi: 10.1007/s11816-019-00581-w

[B72] Kosová K. Vítámvás P. Prášil I. T. (2014). Proteomics of stress responses in wheat and barley-search for potential protein markers of stress tolerance. Front. Plant Sci. 5, 711. doi: 10.3389/fpls.2014.00711 25566285 PMC4263075

[B73] Kouzu M. Hidaka J. (2012). Transesterification of vegetable oil into biodiesel catalyzed by CaO: A review. Fuel 93, 1–12. doi: 10.1016/j.fuel.2011.09.015

[B74] Kretschmer M. Damoo D. Djamei A. Kronstad J. (2019). Chloroplasts and plant immunity: where are the fungal effectors? Pathogens 9 (1), 19. doi: 10.3390/pathogens9010019 31878153 PMC7168614

[B75] Kushanov F. N. Turaev O. S. Ernazarova D. K. Gapparov B. M. Oripova B. B. Kudratova M. K.. (2021). Genetic diversity, QTL mapping, and marker-assisted selection technology in cotton (Gossypium spp.). Front. Plant Sci. 12, 779386. doi: 10.3389/fpls.2021.779386 34975965 PMC8716771

[B76] Lal M. K. Tiwari R. K. Altaf M. A. Kumar A. Kumar R. (2023). Abiotic and biotic stress in horticultural crops: insight into recent advances in the underlying tolerance mechanism. Front. Plant Sci. 14, 1212982. doi: 10.3389/fpls.2023.1212982 37324710 PMC10264795

[B77] Lang Z. Wang Y. Tang K. Tang D. Datsenka T. Cheng J.. (2017). Critical roles of DNA demethylation in the activation of ripening-induced genes and inhibition of ripening-repressed genes in tomato fruit. Proc. Natl. Acad. Sci. U.S.A. 114, E4511–E4519. doi: 10.1073/pnas.1705233114 28507144 PMC5465898

[B78] Lebeda A. Krıstkovaa E. Mieslerovaa B. Dhillonb N. P. S. McCreight J. D. (2024). Status, gaps and perspectives of powdery mildew resistance research and breeding in cucurbits. Crit. Rev. Plant Sci. 43 (4), 211–290. doi: 10.1080/07352689.2024.2315710

[B79] Leida C. Moser C. Esteras C. Sulpice R. Lunn J. E. de Langen F.. (2015). Variability of candidate genes, genetic structure and association with sugar accumulation and climacteric behavior in a broad germplasm collection of melon (Cucumis melo L.). BMC Genet. 16, 28. doi: 10.1186/s12863-015-0183-2 25886993 PMC4380257

[B80] Lemmon Z. H. Bukowski R. Sun Q. Doebley J. F. (2014). The role of *cis* regulatory evolution in maize domestication. PloS Genet. 10, e1004745. doi: 10.1371/journal.pgen.1004745 25375861 PMC4222645

[B81] Li S. Shen L. Hu P. Liu Q. Zhu X. Qian. (2019). Developing diseaseresistant thermosensitive male sterile rice by multiplex gene editing. J. Integr. Plant Biol. 61, 1201–1205. doi: 10.1111/jipb.12774 30623600

[B82] Li J. Wang T. Han J. Ren Z. (2020). Genome-wide identification and characterization of cucumber bHLH family genes and the functional characterization of CsbHLH041 in NaCl and ABA tolerance in Arabidopsis and cucumber. BMC Plant Biol. 20 (1), 272. doi: 10.1186/s12870-020-02440-1 32527214 PMC7291561

[B83] Ling Y. Xiong X. Yang W. Liu B. Shen Y. Xu L.. (2023). Comparative Analysis of Transcriptomics and Metabolomics Reveals Defense Mechanisms in Melon Cultivars against Pseudoperonospora cubensis Infection. Int. J. Mol. Sci. 24, 17552. doi: 10.3390/ijms242417552 38139381 PMC10743968

[B84] Liu J. J. Orlova N. Oakes B. L. Ma E. Spinner H. B. Baney K. L. M.. (2019). CasX enzymes comprise a distinct family of RNA-guided genome editors. Nature 566, 218–223. doi: 10.1038/s41586-019-0908-x 30718774 PMC6662743

[B85] Liu B. Santo Domingo M. Mayobre C. Martín-Hernández A. M. Pujol M. Garcia-Mas J. (2022). Knock-out of CmNAC-NOR affects melon climacteric fruit ripening. Front. Plant Sci. 13, 878037. doi: 10.3389/fpls.2022.878037 35755703 PMC9226586

[B86] Liu Z. Zhu H. Liu Y. Kuang J. Zhou K. Liang F.. (2016). Construction of a high-density, high-quality genetic map of cultivated lotus (Nelumbo nucifera) using next-generation sequencing. BMC Genomics 17, 466. doi: 10.1186/s12864-016-2781-4 27317430 PMC4912719

[B87] Longjan G. G. Dehouche Z. (2018). Nutrient characterization and bioenergy potential of common Nigerian food wastes. Waste Mgt. Res. 36, 426–435. doi: 10.1177/0734242x18763527 29600736

[B88] López-Martıń M. Pérez-de-Castro A. Picó B. Gómez-Guillamón M. L. (2022). Advanced genetic studies on powdery mildew resistance in TGR-1551. Int. J. Mol. Sci. 23 (20), 12553. doi: 10.3390/ijms232012553 36293404 PMC9604395

[B89] Ma J. Li C. Tian J. Qiu Y. Geng L. Wang J. (2023). Identification and fine mapping of gummy stem blight resistance Gene *gsb-7(t)* in melon. Phytopathol. 113, 858–865. doi: 10.1094/PHYTO-05-22-0169-R 35906768

[B90] Madison B. B. Patil D. Richter M. Li X. Cranert S. Wang X.. (2022). Cas-Clover is a novel high-fidelity nuclease for safe and robust generation of TSCM-enriched allogenic CAR-T cells. Mol. Therapy: Nucleic Acids. 29, 979–995. doi: 10.1016/j.omtn.2022.06.003 36189080 PMC9481872

[B91] Malambane G. Madumane K. Sewelo L. T. Batlang U. (2023). Drought stress tolerance mechanisms and their potential common indicators to salinity, insights from the wild watermelon (*Citrullus lanatus*): A review. Front. Plant Sci. 13. doi: 10.3389/fpls.2022.1074395 PMC9939662 36815012

[B92] Malnoy M. Viola R. Jung M. H. Koo O. J. Kim S. Kim J. S.. (2016). DNA free genetically edited grapevine and apple protoplast using CRISPR/Cas9 ribonucleoproteins. Front. Plant Sci. 7, 1904. doi: 10.3389/fpls.2016.01904 28066464 PMC5170842

[B93] Malter D. Wolf S. (2011). Melon phloem-sap proteome: developmental control and response to viral infection. Protoplasma 248, 217–224. doi: 10.1007/s00709-010-0215-8 20924770

[B94] Matsoukas I. G. (2020). Prime editing: genome editing for rare genetic diseases without double-strand breaks or donor DNA. Front. Genet. 11. doi: 10.3389/fgene.2020.00528 PMC7296174 32582281

[B95] Moradpour M. Abdulah S. N. A. (2020). CRISPR/dCas9 platforms in plants: strategies and applications beyond genome editing. Plant Biotechnol. J. 18, 32–44. doi: 10.1111/pbi.13232 31392820 PMC6920162

[B96] Motta M. R. Schnittger A. (2021). A microtubule perspective on plant cell development. Curr. Biol. 31, R547–R552. doi: 10.1016/j.cub.2021.03.087 34033788

[B97] Muhammad B. Y. Mariah A. D. Othman J. B. Yahya A. Bashar Z. U. (2013). [amp]]lsquo;Egusi’ melon (*Citrullus lanatus*) crop – Malaysian new oil/energy source: Production, processing, and prospects. Aust. J. Crop Sci. 7, 2101–2107.

[B98] Mustafa G. Komatsu S. (2021). Plant proteomic research for improvement of food crops under stresses: a review. Mol. Omics 17, 860–880. doi: 10.1039/d1mo00151e 34870299

[B99] National Research Council (2006). “Egusi,” in Lost Crops of Africa: Volume II: Vegetables (The National Academies Press, Washington, DC), 154–171.

[B100] Naeimifar M. Pourrahim R. Zadehdabagh G. (2014). Natural infection of citrullus colocynthis by papaya ringspot virus-w in iran. Plant Dis. 98 (12), 1748. doi: 10.1094/PDIS-06-14-0600-PDN 30703909

[B101] Nieto C. Morales M. Orjeda G. Clepet C. Monfort A. Sturbois B.. (2006). An eIF4E allele confers resistance to an uncapped and non- polyadenylated RNA virus in melon. Plant J. 4, 452–462. doi: 10.1111/j.1365-313X.2006.02885.x 17026540

[B102] Nizan S. Amitzur A. Dahan-Meir T. Benichou J. I. C. Bar-Ziv A. Perl-Treves R. (2023). Mutagenesis of the melon Prv gene by CRISPR/Cas9 breaks papaya ringspot virus resistance and generates an autoimmune allele with constitutive defense responses. J. Exp. Bot. 74, 4579–4596. doi: 10.1093/jxb/erad156 37137337 PMC10433930

[B103] Nonaka S. Ezura H. (2024). Possible of genome editing for melon breeding. Breed. Sci. 74 (1), 47–58. doi: 10.1270/jsbbs.23074 39246433 PMC11375426

[B104] Nonaka S. Ito M. Ezura H. (2023). Targeted modification of CmACO1 by CRISPR/Cas9 extends the shelf-life of Cucumis melo var. reticulatus melon. Front. Genome Ed. 5, 1176125. doi: 10.3389/fgeed.2023.1176125 37304010 PMC10249633

[B105] Ntui V. O. Khan R. S. Chin D. P. Nakamura I. Mii M. (2010a). An efficient *Agrobacterium tumefaciens*-mediated genetic transformation of “’Egusi’ melon (*Colocynthisis citrullus* L.). Plant Cell Tissue Organ Culture 103, 15–22. doi: 10.1007/s11240-010-9748-y

[B106] Ntui V. O. Samwel K. M. Tripathi N. J. Tripathi L. (2023). Cassava molecular genetics and genomics for enhanced resistance to diseases and pests. Mol. Plant Pathol. 25, e13402. doi: 10.1111/mpp.13402 37933591 PMC10788594

[B107] Ntui V. O. Thirukkumaran G. Iioka S. Mii M. (2009). Efficient plant regeneration via organogenesis in “ ‘Egusi’ melon (*Colocynthis citrullus* L.). Scientia Hortic. 119, 397–402. doi: 10.1016/j.scienta.2008.08.031

[B108] Ntui V. O. Thirukkumarn G. Azadi P. Khan R. S. Chin D. P. Nakamura I.. (2010b). Stable integration and expression of wasabi defensin gene in “’Egusi’ melon (*Colocynthis citrullus* L.) confers resistance to Fusarium wilt and Alternaria leaf spot. Plant Cell Rep. 29, 943–954. doi: 10.1007/s00299-010-0880-2 20552202

[B109] Ntui V. O. Tripathi J. N. Shah T. Tripathi L. (2024). Targeted knockout of early nodulin-like 3 (MusaENODL3) gene in banana reveals its function in resistance to Xanthomonas wilt disease. Plant Biotechnol. J. 22, 1101–1112. doi: 10.1111/pbi.14248 38013635 PMC11022791

[B110] Ntui V. O. Uyoh E. A. (2004). Intraspecific hybridization in ‘Egusi’ melon, *Colocynthis citrullus* L. Glo. J. Pure Appl. Sci. 10, 519–523. doi: 10.4314/gjpas.v10i4.16434

[B111] Ntui V. O. Uyoh E. A. (2005). Inheritance of stripe pattern on fruits and Seed colour in ‘Egusi’ melon, *Colocynthis citrullus* L. Glo. J. Agri. Sci. 4, 29–32. doi: 10.4314/gjass.v4i1.2251

[B112] Nwakaudu A. A. Nwakaudu M. S. Owuamanam C. I. Alagbaoso S. O. Njoku N. E. Agunwah I. M.. (2017). Ascertaining the shelf-life of ground melon seed (*Cococynthis citrullus*). Eur. J. Food Sci. Technol. 5, 13–21.

[B113] Nwoke S. I. Okechukwu Q. N. Ugwuona F. U. Ojukwu M. Skendrović H. Juchniewicaz S.. (2023). Flour nutritional profile, and soxhlet-extracted oil physicochemical breakdown-storage performance of white melon (*Cucumeropsis mannii* Naudin) seed varieties from Southeast Nigeria. PloS One 18, e0282974. doi: 10.1371/journal.pone.0282974 37167260 PMC10174525

[B114] Nyakuma B. (2017). Bioenergy potential of melon seed husks. Lignocellulose 6, 15–22.

[B115] Nzelu C. O. Okonkwo N. J. (2016). Evaluation of melon seed oil *Citrullus colocynthis* (L.) Schrad, for the protection of cowpea *Vigna unguiculata* seeds against *Callosobruchus maculatus* (Fabricius) (Coleoptera: Bruchidae). Evaluation 3, 1–10. doi: 10.17148/iarjset.2016.3813

[B116] Ogbu V. O. Ogbonna P. Onyia V. Okechukwu E. (2016). Yield improvement of ‘Egusi’’ melon *(Colocynthis citrillus L.)* through intergeneric hybridization with watermelon *(Citrillus lanatus L.)* . Available online at: http://www.thejaps.org.pk/docs/v-26-05/14.pdf (Accessed June 16, 2024).

[B117] Ogundare S. A. Moodley V. Amaku J. F. Ogunmoye A. O. Atewolara-Odule O. C. Olubomehin O. O.. (2021). Nanocrystalline cellulose derived from melon seed shell (Citrullus colocynthis L.) for reduction and stabilization of silver nanoparticles: Synthesis and catalytic activity. Carbohydr. Polymer Technol. Appl. 2, 100134. doi: 10.1016/j.carpta.2021.100134

[B118] Ogunsola J. F. Ikotun B. Ogunsola K. E. (2020). Incidence of leaf blight disease of ‘Egusi’ melon in South-west Nigeria. Afr. Crop Sci. J. 28, 255–265. doi: 10.4314/acsj.v28i2.10

[B119] Oji U. I. Oruwari B. M. Iwuagila R. O. (1999). Performance of growing broiler chickens fed toasted and untoasted melon (Colocynthis citrullus) seed meal. Trop. J. Anim. Sci. 1, 43–49. doi: 10.4314/tjas.v1i1.49530

[B120] Oke E. O. Adeyi O. Okolo B. I. Ude C. J. Adeyi J. A. Salam K. K.. (2021). Heterogeneously catalyzed biodiesel production from Azadirachta indica oil: Predictive modelling with uncertainty quantification, experimental optimization, and techno-economic analysis. Bioresour. Technol. 332, 125141. doi: 10.1016/j.biortech.2021.125141 33862384

[B121] Okwundu O. S. Chiama C. J. Chima C. J. Ucheagwu P. C. Uzoma E. K. Okaro C. A.. (2021). The untapped industrial crop, *Cucumeropsis mannii*: dry oil extraction, characterization, and potential use as biodiesel feedstock and heavy metal sink. Sustain. Environ. Res. 31, 10. doi: 10.1186/s42834-021-00082-y

[B122] Oladapo O. O. Bolaji M. O. Abdul-Malik O. (2017). Comparative study of artherogenic effects of common Nigerian edible oils in male rabbits. J. Adv. Med. Med. Res. 20, 1–10. doi: 10.9734/bjmmr/2017/32060

[B123] Oloyede O. B. Otunola G. A. Apata D. F. (2004). Assessment of protein quality of processed melon seed as a component of poultry feed. Biokemistri 16, 80–87. doi: 10.4314/biokem.v16i2.32574

[B124] Olubi O. Felix-Minnaar J. V. Jideani V. A. (2019). Physicochemical and fatty acid profile of ‘Egusi’ oil from supercritical carbon dioxide extraction. Heliyon 5, e01083. doi: 10.1016/j.heliyon.2018.e01083 30619961 PMC6313835

[B125] Olubi O. Felix-Minnaar J. V. Jideani V. A. (2021). Physicochemical, Mineral and Sensory Characteristics of Instant Citrullus lanatus *mucosospermus* (‘Egusi’*)* Soup. Foods 10, 1817. doi: 10.3390/foods10081817 34441594 PMC8391701

[B126] Opoku-Boahen Y. Clemente R. Wubah D. (2013). Investigation of *Cucumeropsis mannii* N. seed oil as potential biodiesel feedstock. Int. J. Biol. Chem. Sci. 7, 1767–1761. doi: 10.4314/ijbcs.v7i4.32

[B127] Oren E. Dafna A. Tzuri G. Halperin I. Isaacson T. Elkabetz M.. (2022). Pan-genome and multi-parental framework for high-resolution trait dissection in melon (Cucumis melo). Plant J.: For Cell Mol. Biol. 112, 1525–1542. doi: 10.1111/tpj.16021 PMC10100132 36353749

[B128] Orji V. O. Ogbonna P. E. Onyia V. N. Okechukwu E. C. (2016). Improvement of fruit taste quality of ‘Egusi’ melon (*Colocynthis citrullus* L.) through hybridization with watermelon (Citrullus lanatus Thumb.). J. Trop. Agric. 54, 1–1.

[B129] Ortigosa A. Gimenez-Ibanez S. Leonhardt N. Solano R. (2019). Design of a bacterial speck resistant tomato by CRISPR/Cas9-mediated editing of SlJAZ2. Plant Biotechnol. J. 17, 665–673. doi: 10.1111/pbi.13006 30183125 PMC6381780

[B130] Owolabi R. U. Usman M. A. Wisdom C. E. (2020). Trans-esterification of castor oil using heterogeneous biocatalyst obtained from bovine bone (Romania: Acta Technica Corviniensis – Bulletin of Engineering). TOME XIII [2020] | FASCICULE 2 [April – June].

[B131] Pal S. Rao E. S. Reddy D. CL. and Dayanandhi E. (2023). QTL-seq identifies and QTL mapping refines genomic regions conferring resistance to fusarium oxysporum f. sp. niveum race 2 in cultivated watermelon [Citrullus lanatus (Thunb.) matsum & nakai]. Sci. Horti. 319, 112180. doi: 10.1016/j.scienta.2023.112180

[B132] Pane C. Sorrentino R. Scotti R. Molisso M. Di Matteo A. Celano G.. (2020). Alpha and beta-diversity of microbial communities associated to plant disease suppressive functions of on-farm green composts. Agriculture 10 (4), 113. doi: 10.3390/agriculture10040113

[B133] Paris H. S. (2015). Origin and emergence of the sweet dessert watermelon, Citrullus lanatus. Ann. Bot. 116, 133–148. doi: 10.1093/aob/mcv077 26141130 PMC4512189

[B134] Paris M. K. Zalapa J. E. McCreight J. D. Staub J. E. (2008). Genetic dissection of fruit quality components in melon (Cucumis melo L.) using a RIL population derived from exotic × elite US Western Shipping germplasm. Mol. Breeding: New Strategies Plant Improvement 22, 405–419. doi: 10.1007/s11032-008-9185-3

[B135] Park J.-J. Dempewolf E. Zhang W. Wang Z.-Y. (2017). RNA-guided transcriptional activation via CRISPR/dCas9 mimics overexpression phenotypes in Arabidopsis. PloS One 12, e0179410. doi: 10.1371/journal.pone.0179410 28622347 PMC5473554

[B136] Pascual L. Yan J. Pujol M. Monforte A. J. Picó B. Martín-Hernández A. M. (2019). CmVPS41 is a general gatekeeper for resistance to cucumber mosaic virus phloem entry in melon. Front. Plant Sci. 10, 1219. doi: 10.3389/fpls.2019.01219 31632432 PMC6781857

[B137] Patel T. Quesada-Ocampo L. M. Wehner T. C. Bhatta B. P. Correa E. Malla S. (2023). Recent advances and challenges in management of colletotrichum orbiculare, the causal agent of watermelon anthracnose. Hortic. 9 (10), 1132. doi: 10.3390/horticulturae9101132

[B138] Paudel L. Clevenger J. McGregor C. (2019). Chromosomal locations and interactions of four loci associated with seed coat color in watermelon. Front. Plant Sci. 10. doi: 10.3389/fpls.2019.00788 PMC6603093 31293604

[B139] Pereira L. Santo Domingo M. Argyris J. Mayobre C. Valverde L. Martín-Hernández A. M.. (2021). A novel introgression line collection to unravel the genetics of climacteric ripening and fruit quality in melon. Sci. Rep. 11, 11364. doi: 10.1038/s41598-021-90783-6 34059766 PMC8166866

[B140] Pérez-Escobar O. A. Tusso S. Przelomska N. A. S. Wu S. Ryan P. Nesbitt M.. (2022). Genome sequencing of up to 6,000-year-old citrullus seeds reveals use of a bitter-fleshed species prior to watermelon domestication. Mol. Biol. Evol. 39, msac168. doi: 10.1093/molbev/msac168 35907246 PMC9387916

[B141] Pérez-de-Castro A. Esteras C. Alfaro-Fernández A. Dáros J. Monforte A. J. Picó B.. (2019). Fine mapping of *wmv^1551^ *, a resistance gene to *Watermelon mosaic virus* in melon. Mol. Breed. 39, 93. doi: 10.1007/s11032-019-0998-z

[B142] Pichot C. Djari A. Tran J. Verdenaud M. Marande W. Huneau C.. (2022). Cantaloupe melon genome reveals 3D chromatin features and structural relationship with the ancestral cucurbitaceae karyotype. iScience 25, 103696. doi: 10.1016/j.isci.2021.103696 35059606 PMC8760558

[B143] Polonio Á. Pineda M. Bautista R. Martínez-Cruz J. Pérez-Bueno M. L. Barón M.. (2019). RNA-seq analysis and fluorescence imaging of melon powdery mildew disease reveal an orchestrated reprogramming of host physiology. Sci. Rep. 9, 7978. doi: 10.1038/s41598-019-44443-5 31138852 PMC6538759

[B144] Ponsankar A. Senthil-Nathan S. Vasantha-Srinivasan P. Pandiyan R. Karthi S. Kalaivani K.. (2023). Systematic induced resistance in solanum lycopersicum (L.) against vascular wilt pathogen (Fusarium oxysporum f. sp. lycopersici) by citrullus colocynthis and trichoderma viride. PloS One 18 (5), e0278616. doi: 10.1371/journal.pone.0278616 37130086 PMC10153711

[B145] Prothro J. Sandlin K. Gill R. (2012). Mapping of the egusi seed trait locus (eg) and quantitative trait loci associated with seed oil percentage in watermelon. J. Amer. Soc Hortic. Sci. 137 (5), 311–315.

[B146] Qi L. S. Larson M. H. Gilbert L. A. Doudna J. A. Weissman J. S. Arkin A. P.. (2013). Repurposing CRISPR as an RNA-guided platform for sequence-specific control of gene expression. Cell 152, 1173–1183. doi: 10.1016/j.cell.2013.02.022 23452860 PMC3664290

[B147] Rahman K. U. Ejaz N. Shang S. Balkhair K. S. Alghamdi K. Zaman K. (2024). A robust integrated agricultural drought index under climate and land use variations at the local scale in pakistan. Agric. Water Manage. 295, 108748. doi: 10.1016/j.agwat.2024.108748

[B148] Real N. Villar I. Serrano I. Guiu-Aragonés C. Martín-Hernández A. M. (2023). Mutations in CmVPS41 controlling resistance to cucumber mosaic virus display specific subcellular localization. Plant Physiol. 191 (3), 1596–1611. doi: 10.1093/plphys/kiac583 36527697 PMC10022621

[B149] Reddy A. N. R. Saleh A. A. Islam M. S. Hamdan S. MAleque M. A. (2016). Biodiesel production from crude Jatropha oil using a highly active heterogeneous nanocatalyst by optimizing transesterification reaction parameters. Energy Fuels 30, 334–343. doi: 10.1021/acs.energyfuels.5b01899

[B150] Rodríguez-Moreno L. González V. M. Benjak A. Martí M. C. Puigdomènech P. Aranda M. A.. (2011). Determination of the melon chloroplast and mitochondrial genome sequences reveals that the largest reported mitochondrial genome in plants contains a significant amount of DNA having a nuclear origin. BMC Genomics 12, 424. doi: 10.1186/1471-2164-12-424 21854637 PMC3175227

[B151] Roig C. Fita A. Ríos G. Hammond J. P. Nuez F. Picó B. (2012). Root transcriptional responses of two melon genotypes with contrasting resistance to Monosporascus cannonballus (Pollack et Uecker) infection. BMC Genomics 13, 601. doi: 10.1186/1471-2164-13-601 23134692 PMC3542287

[B152] Roy A. Dutta S. Das S. Choudhury M. R. (2024). Next-generation sequencing in the development of climate-resilient and stress-responsive crops – A review. Open Biotechnol. J. 18, 6036. doi: 10.2174/0118740707301657240517063244

[B153] Ruggieri V. Alexiou K. G. Morata J. Argyris J. Pujol M. Yano R.. (2018). An improved assembly and annotation of the melon (*Cucumis melo* L.) reference genome. Sci. Rep. 8, 8088. doi: 10.1038/s41598-018-26416-2 29795526 PMC5967340

[B154] Saitoh H. Kiba A. Nishihara M. Yamamura S. Suzuki K. Terauchi R. (2001). Production of antimicrobial defensin in *Nicotiana benthamiana* with a potato virus 9 vector. Mol. Plant Microbe Interact. 14, 111–115. doi: 10.1094/MPMI.2001.14.2.111 11204773

[B155] Saladié M. Cañizares J. Phillips M. A. Rodriguez-Concepcion M. Larrigaudière C. Gibon Y.. (2015). Comparative transcriptional profiling analysis of developing melon (Cucumis melo L.) fruit from climacteric and non-climacteric varieties. BMC Genomics 16, 440. doi: 10.1186/s12864-015-1649-3 26054931 PMC4460886

[B156] Salekdeh G. H. Komatsu S. (2007). Crop proteomics: aim at sustainable agriculture of tomorrow. Proteomics 7, 2976–2996. doi: 10.1002/pmic.200700181 17639607

[B157] Santo Domingo M. Areco L. Mayobre C. Valverde L. Martín-Hernández A. M. Pujol M.. (2022). Modulating climacteric intensity in melon through QTL stacking. Hortic. Res. 9, uhac131. doi: 10.1093/hr/uhac131 35928400 PMC9343914

[B158] Santos S. Nobre L. Gomes J. Puna J. Quinta-Ferreira R. Bordado J. (2019). Soybean oil transesterification for biodiesel production with micro-structured calcium oxide (CaO) from natural waste materials as a heterogeneous catalyst. Energies 12, 467. doi: 10.3390/en12244670

[B159] Sanz-Carbonell A. Marques M. C. Martinez G. Gomez G. (2020). Dynamic architecture and regulatory implications of the miRNA network underlying the response to stress in melon. RNA Biol. 17, 292–308. doi: 10.1080/15476286.2019.1697487 31766933 PMC6973316

[B160] Sasnauskas G. Tamulaitiene G. Druteika G. Carabias A. Silanskas A. Kazlauskas D.. (2023). TnpB structure reveals minimal functional core of Cas12 nuclease family. Nature 616, 384–389. doi: 10.1038/s41586-023-05826-x 37020015

[B161] Schippers R. R. (2000). African indigenous vegetables: An overview of the cultivated species (Chatham, UK: Natural Resources Institute/ACP-EU Technical Centre for Agricultural and Rural Cooperation).

[B162] Serra-Soriano M. Navarro J. A. Genoves A. Pallás V. (2015). Comparative proteomic analysis of melon phloem exudates in response to viral infection. J. Proteomics 124, 11–24. doi: 10.1016/j.jprot.2015.04.008 25892132

[B163] Seymour G. B. Granell A. (2014). Fruit development and ripening. J. Exp. Bot. 65, 4489–4490. doi: 10.1093/jxb/eru307 25221812 PMC4115256

[B164] Shao X. Liu F. Shen Q. He W. Jia B. Fan Y.. (2024). Transcriptomics and metabolomics reveal major quality regulations during melon fruit development and ripening. Food Innovation Adv. 3, 144–154. doi: 10.48130/fia-0024-0013

[B165] Shi X. Wang X. Cheng F. Cao H. Liang H. Lu J.. (2019). iTRAQ-based quantitative proteomics analysis of cold stress-induced mechanisms in grafted watermelon seedlings. J. Proteom. 192, 311–320. doi: 10.1016/j.jprot.2018.09.012 30267873

[B166] Shirazi Parsa H. Sabet M. S. Moieni A. Shojaeiyan A. Dogimont C. Boualem A.. (2023). CRISPR/Cas9-mediated cytosine base editing using an improved transformation procedure in melon (Cucumis melo L.). Int. J. Mol. Sci. 24, 11189. doi: 10.3390/ijms241311189 37446368 PMC10342833

[B167] Sim S.-C. Nguyen N. N. Kim N. Kim J. Park Y. (2018). Whole-genome resequencing reveals genome-wide single nucleotide polymorphisms between orange-fleshed and green-fleshed melons. Hortic. Environ. Biotechnol. 59, 275–283. doi: 10.1007/s13580-018-0030-2

[B168] Song Q. Joshi M. Joshi V. (2020). Transcriptomic analysis of short-term salt stress response in watermelon seedlings. Int. J. Mol. Sci. 21 (17), 6036. doi: 10.3390/ijms21176036 32839408 PMC7504276

[B169] Song W. Tang F. Cai W. Zhang Q. Zhou F. Ning M.. (2020). iTRAQ-based quantitative proteomics analysis of cantaloupe (Cucumis melo var. saccharinus) after cold storage. BMC Genomics 21, 390. doi: 10.1186/s12864-020-06797-3 32493266 PMC7268308

[B170] Song W. Zhou F. Shan C. Zhang Q. Ning M. Liu X.. (2021). Identification of Glutathione S-Transferase Genes in Hami Melon (Cucumis melo var. saccharinus) and Their Expression Analysis Under Cold Stress. Front. Plant Sci. 12, 672017. doi: 10.3389/fpls.2021.672017 34168669 PMC8217883

[B171] Suleiman I. Y. Salihu S. A. Mohammed T. A. (2018). Investigation of mechanical, microstructure, and wear behaviors of Al-12%Si reinforced with melon shell ash particulates. Int. Adv. Manuf. Technol. 97, 4137–4144. doi: 10.1007/s00170-018-2157-9

[B172] Sultana R. S. Rahman M. M. (2013). Biotechnological approaches of watermelon to meet the future challenges for next decades. Adv. Biosci. Bioeng. 1, 40–48. doi: 10.11648/j.abb.20130102.11

[B173] Swinnen G. Goossens A. Pauwels L. (2016). Lessons from domestication: targeting cis-regulatory elements for crop improvement. Trends Plant Sci. 21, 506–515. doi: 10.1016/j.tplants.2016.01.014 26876195

[B174] Tahvildari K. Chitsaz H. R. Mozaffarinia P. (2014). Heterogeneous catalytic modified process in the production of biodiesel from sunflower oil, waste cooking oil and olive oil by transesterification method. Acad. Res. Int. 5, 60–68.

[B175] Takikawa Y. Nonomura T. Miyamoto S. Okamoto N. Murakami T. Matsuda Y.. (2015). Digital microscopic analysis of conidiogenesis of powdery mildew pathogens isolated from melon leaves. Phytoparasitica; Israel J. Plant Prot. Sci. 43, 517–530. doi: 10.1007/s12600-015-0467-0

[B176] Tao H. Shi X. He F. Wang D. Xiao N. Fang H.. (2021). Engineering broad-spectrum diseaseresistant rice by ed-iting multiple susceptibility genes. J. Integr. Plant Biol. 63, 1639–1648. doi: 10.1111/jipb.13145 34170614

[B177] Tettelin H. Medini D. (2020). The Pangenome: Diversity, Dynamics and Evolution of Genomes (Gewerbestrasse 11, Cham, Ch 6330, Switzerland: Springer Nature). 32633908

[B178] Thangavel P. Natarajan S. Shanmugam V. Sulaiman S. H. Ramasamy R. (2019). Lofty frequency and reproducible plant regeneration from mature nodal explants of ‘Egusi’ melon (*Citrullus colocynthis* L.). BioTechnologia 100, 263–272. doi: 10.5114/bta.2019.87585

[B179] Thevissen K. Terras F. R. G. Broekaert W. F. (1999). Permeabilization of fungal membranes by plant defensins inhibits fungal growth. Appl. Environ. Microbiol. 65, 5451–5458. doi: 10.1128/aem.65.12.5451-5458.1999 10584003 PMC91743

[B180] Thomazella D. P. T. Seong K. Mackelprang R. Dahlbeck D. Geng Y. Gill U. S.. (2021). Loss of function of a DMR6 ortholog in tomato confers broad-spectrum disease resistance. Proc. Natl. Acad. Sci. U. S. A. 118, e2026152118. doi: 10.1073/pnas.2026152118 34215692 PMC8271637

[B181] Tian S. Jiang L. Cui X. Zhang J. Guo S. Li M.. (2018). Engineering herbicide-resistant watermelon variety through CRISPR/Cas9-mediated base-editing. Plant Cell Rep. 37, 1353–1356. doi: 10.1007/s00299-018-2299-0 29797048

[B182] Tian S. Jiang L. Gao Q. Zhang J. Zong M. Zhang H.. (2017). Efficient CRISPR/Cas9-based gene knockout in watermelon. Plant Cell Rep. 36, 399–406. doi: 10.1007/s00299-016-2089-5 27995308

[B183] Tripathi J. N. Ntui V. O. Shah T. Tripathi L. (2021). CRISPR/Cas9-mediated editing of DMR6 orthologue in banana (Musa spp.) confers enhanced resistance to bacterial disease. Plant Biotechnol. J. 19, 1291–1293. doi: 10.1111/pbi.13614 33934462 PMC8313124

[B184] Tripathi L. Ntui V. O. Tripathi J. N. (2020). CRISPR/Cas9-based genome editing of banana for disease resistance. Curr. Opin. Plant Biol. 56, 118–126. doi: 10.1016/j.pbi.2020.05.003 32604025

[B185] Tripathi L. Ntui V. O. Tripathi J. N. (2024). Application of CRISPR/Cas-based gene-editing for developing better banana. Front. Bioeng. Biotechnol. 12. doi: 10.3389/fbioe.2024.1395772 PMC11362101 39219618

[B186] Uday G. Shardha H. B. Priyanka K. Jagirdhar S. (2024). “Advances in plant proteomics toward improvement of crop productivity and stress resistance,” in Plant Proteomics (Boca Raton, Florida, United States: CRC Press), 11–29.

[B187] Uruakpa F. O. Aluko R. E. (2004). Heat-induced gelation of whole ‘Egusi’ (*Colocynthis citrullus* L.) seeds. Food Chem. 87, 349–354. doi: 10.1016/j.foodchem.2003.12.005

[B188] Uzogara S. G. Agu L. N. Uzogara E. O. (1990). A review of traditional fermented foods, condiments and bevergaes in nigeria: Their benefits and possible problems. Ecol. Food Nutr. 24 (4), 267–288. doi: 10.1080/03670244.1990.9991145

[B189] Wallace E. C. Quesada-Ocampo L. M. (2017). Analysis of microsatellites from the transcriptome of downy mildew pathogens and their application for characterization of pseudoperonospora populations. PeerJ 5, e3266. doi: 10.7717/peerj.3266 28480143 PMC5417063

[B190] Wan S. Truong-Trieu V. M. T. Ward T. Whalen J. K. Altosaar I. (2017). Advances in the use of genetically modified plant biomass for biodiesel generation. Biofuels Bioprod. Bioref. 11, 749–764. doi: 10.1002/bbb.1777

[B191] Wang N. Fang L. Xin H. Wang L. Li S. (2012). Construction of a high-density genetic map for grape using next generation restriction-site associated DNA sequencing. BMC Plant Biol. 12, 148. doi: 10.1186/1471-2229-12-148 22908993 PMC3528476

[B192] Wang Z. Yadav V. Chen X. Zhang S. Yuan X. Li H.. (2023). Multi-omics analysis reveals intricate gene networks involved in female development in melon. Int. J. Mol. Sci. 24 (23), 16905. doi: 10.3390/ijms242316905 38069227 PMC10706797

[B193] Wang Y. Zhou J. Wen W. Sun J. Shu S. Guo S. (2023). Transcriptome and proteome analysis identifies salt stress response genes in bottle gourd rootstock-grafted watermelon seedlings. Agronomy 13, 618. doi: 10.3390/agronomy13030618

[B194] Wei M. Huang Y. Mo C. Wang H. Zeng Q. Yang W.. (2023). Telomere-to-telomere genome assembly of melon (*Cucumis melo* L. var. inodorus) provides a high-quality reference for meta-QTL analysis of important traits. Hortic. Res. 10, uhad189. doi: 10.1093/hr/uhad189 37915500 PMC10615816

[B195] Wu P. Ye X. Wang D. Gong F. Wei X. Xiang S.. (2022). A novel CRISPR/Cas14a system integrated with 2D porphyrin metal-organic framework for microcystin-LR determination through a homogeneous competitive reaction. J. Hazard Mater. 15;424, 127690. doi: 10.1016/j.jhazmat.2021.127690 34799170

[B196] Xiang G. Li Y. Sun J. Huo Y. Cao S. Cao. (2023). Evolutionary mining and functional characterization of TnpB nucleases identify efficient miniature genome editors. Nat. Biotechnol. 42 (5), 745–757. doi: 10.1038/s41587-023-01857-x 37386294

[B197] Xu Y. Li P. Yang Z. Xu C. (2017). Genetic mapping of quantitative trait loci in crops. Crop J. 5, 175–184. doi: 10.1016/j.cj.2016.06.003

[B198] Yang H. Patel D. J. (2019). CasX: a new and small CRISPR gene-editing protein. Cell Res. 29, 345–346. doi: 10.1038/s41422-019-0165-4 30992542 PMC6796933

[B199] Yang L. Machin F. Wang S. Saplaoura E. Kragler F. (2023). Heritable transgene-free genome editing in plants by grafting of wild-type shoots to transgenic donor rootstocks. Nat. Biotechnol. 41, 958–967. doi: 10.1038/s41587-022-01585-8 36593415 PMC10344777

[B200] Yang Y. Saand M. A. Huang L. Abdelaal W. B. Zhang J. Wu Y.. (2021). Applications of multi-omics technologies for crop improvement. Front. Plant Sci. 12, 563953. doi: 10.3389/fpls.2021.563953 34539683 PMC8446515

[B201] Yano R. Nonaka S. Ezura H. (2018). Melonet-DB, a grand RNA-Seq gene expression atlas in melon (Cucumis melo L.). Plant Cell Physiol. 59, e4. doi: 10.1093/pcp/pcx193 29216378

[B202] Yano R. Ariizumi T. Nonaka S. Kawazu Y. Zhong S. Mueller L. (2020). Comparative genomics of muskmelon reveals a potential role for retrotransposons in the modification of gene expression. Commun. Biol. 3, 432. doi: 10.1038/s42003-020-01172-0 32792560 PMC7426833

[B203] Yoshimura K. Masuda A. Kuwano M. Yokota A. Akashi K. (2008). Programmed proteome response for drought avoidance/tolerance in the root of a C(3) xerophyte (wild watermelon) under water deficits. Plant Cell Physiol. 49, 226–241. doi: 10.1093/pcp/pcm180 18178965

[B204] Yu Y. Guo S. Ren Y. Zhang J. Li M. Tian S.. (2022). Quantitative transcriptomic and proteomic analysis of fruit development and ripening in watermelon (Citrullus lanatus). Front. Plant Sci. 13, 818392. doi: 10.3389/fpls.2022.818392 35392508 PMC8980866

[B205] Yu Q. Powles S. B. (2014). Resistance to AHAS inhibitor herbicides: current understanding. Pest Manag. Sci. 70, 1340–1350. doi: 10.1002/ps.3710 24338926

[B206] Yu J. Wu S. Sun H. Wang X. Tang X. Guo S.. (2023). CuGenDBv2: An updated database for cucurbit genomics. Nucleic Acids Res. 51, D1457–D1464. doi: 10.1093/nar/gkac921 36271794 PMC9825510

[B207] Zhang X. Ling Y. Yang W. Wei M. Wang Z. Li M.. (2023). Fine mapping of a novel QTL *DM9.1* conferring downy mildew resistance in melon. Front. Plant Sci. 14. doi: 10.3389/fpls.2023.1202775 PMC10291176 37377806

[B208] Zhang M. Liu Q. Yang X. Xu J. Lui G. Yao X.. (2020). CRISPR/Cas9-mediated mutagenesis of *Clpsk1* in watermelon to confer resistance to *Fusarium oxysporum* f.sp. niveum. Plant Cell Rep. 39, 589–595. doi: 10.1007/s00299-020-02516-0 32152696

[B209] Zhang (2020). QTL mapping of pericarp and fruit-related traits in melon (Cucumis melo L.) using SNP-derived CAPS markers. Scientia Hortic. 265, 109243. doi: 10.1016/j.scienta.2020.109243

[B210] Zhang H. Wang H. Yi H. Zhai W. Wang G. Fu Q. (2016). Transcriptome profiling of Cucumis melo fruit development and ripening. Hortic. Res. 3, 16014. doi: 10.1038/hortres.2016.14 27162641 PMC4847005

[B211] Zhao Z. Dong Y. Wang J. Zhang G. Zhang Z. Zhang A.. (2022). Comparative transcriptome analysis of melon (Cucumis melo L.) reveals candidate genes and pathways involved in powdery mildew resistance. Sci. Rep. 12, 4936. doi: 10.1038/s41598-022-08763-3 35322050 PMC8943038

[B212] Zhou J. Peng Z. Long J. Sosso D. Liu B. Eom J. S.. (2015). Gene targeting by the TAL effector PthXo2 reveals cryptic resistance gene for bacterial blight of rice. Plant J. 82, 632–643. doi: 10.1111/tpj.12838 25824104

[B213] Zhou B. Yang R. Sohail M. Kong X. Zhang X. Fu N.. (2023). CRISPR/Cas14 provides a promising platform in facile and versatile aptasensing with improved sensitivity. Talanta 254, 124120. doi: 10.1016/j.talanta.2022.124120 36463799

